# Supplementary material for: A Novel Assay for Profiling GBM Cancer Model Heterogeneity and Drug Screening
Source: Cells. 2019 Jul 11;8(7):702. doi: 10.3390/cells8070702 (PMC6678976; doi:10.3390/cells8070702)
Supplement: Supplementary file 1 [file cells-08-00702-s001.zip › Supplemental Figure Legends.pdf]

Figure S1. Confusion matrix of classifier accuracy using random forest classifiers of 100 features selected by FastEMC. Five-fold stratified cross validation was used for predictions. Accurate sample classifications are counted on the diagonal. Inaccurate classifications are counted off the diagonal. i.e. 3 Classical samples were misclassified as Proneural.

Figure S2. Plot of log-distance to medoid. K-medoids were calculated for each of the 4 subtypes and the log-distance of individual samples to its respective classifier medoid was measured using the 100 features selected from FastEMC. A smaller distance to the medoid is predictive of class membership to that subtype.

Figure S3. Hierarchically clustered heatmap of samples based on FastEMC gene features. FastEMC gene features cluster GBM-PDX samples by tumor subtype revealing patterns of gene expression specific to each subtype.

Table S1. NanoString Chip Signatures

Table S2. Genes Associated with Temozolomide Response

Supplementary File 1. Drug Response Slides

Supplementary File 2. Affymetrix Data (Normalized)

Supplementary File 3. NanoString Data (Normalized)
