# Supplementary material for: A Novel Assay for Profiling GBM Cancer Model Heterogeneity and Drug Screening
Source: Cells. 2019 Jul 11;8(7):702. doi: 10.3390/cells8070702 (PMC6678976; doi:10.3390/cells8070702)

## Slide 1
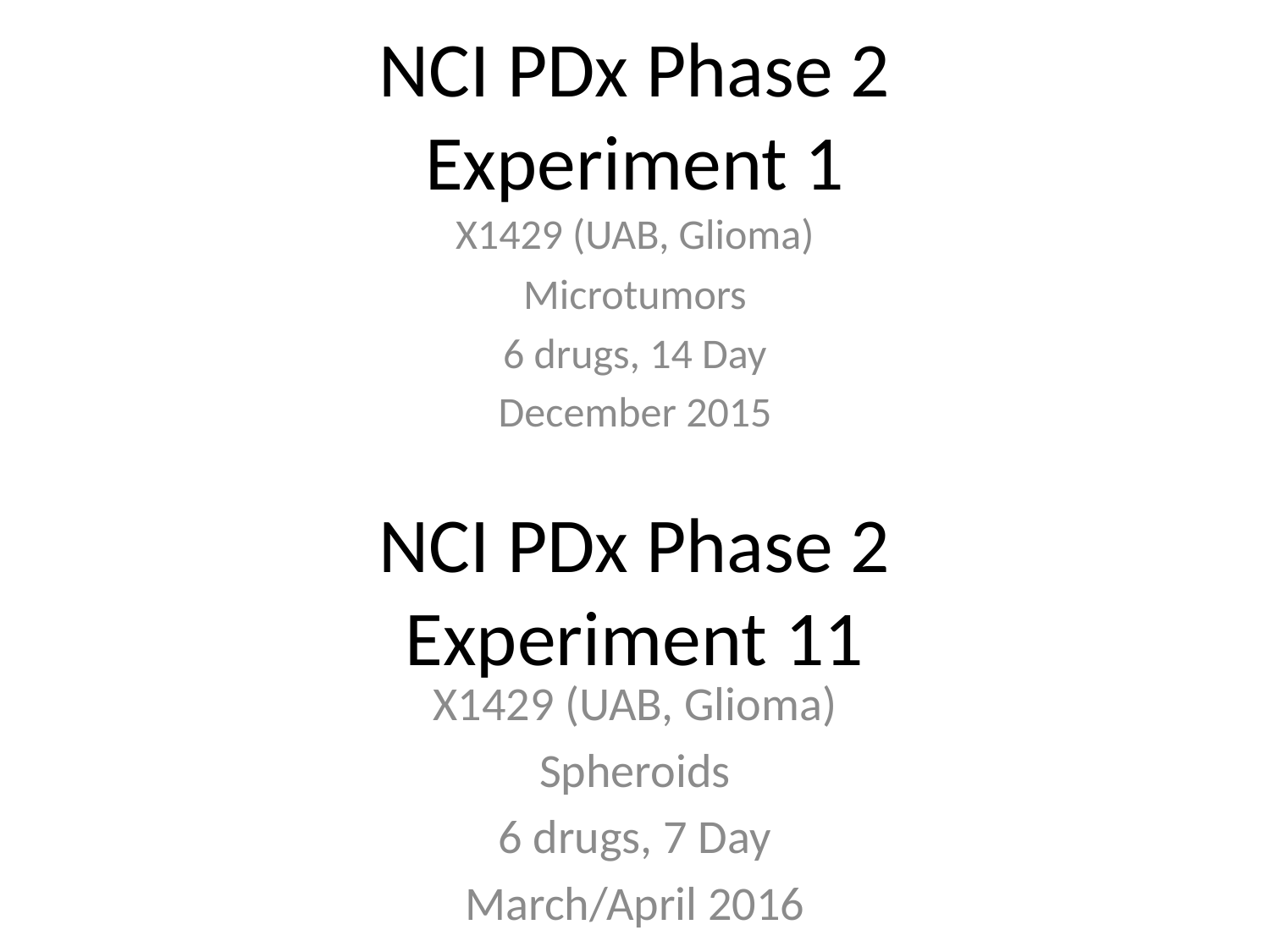

# NCI PDx Phase 2 Experiment 1
X1429 (UAB, Glioma)
Microtumors
6 drugs, 14 Day
December 2015
NCI PDx Phase 2
Experiment 11
X1429 (UAB, Glioma)
Spheroids
6 drugs, 7 Day
March/April 2016

## Slide 2
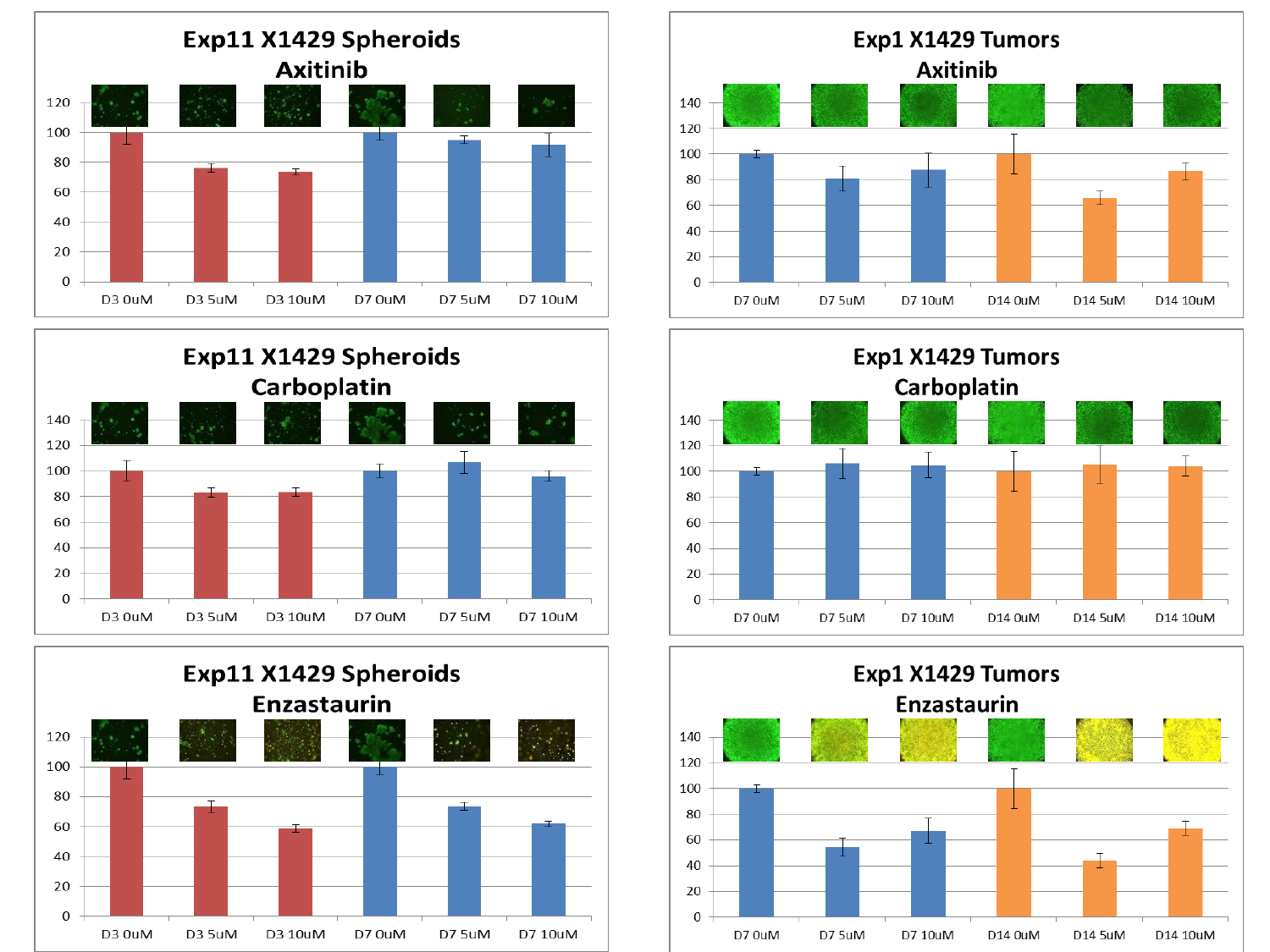

## Slide 3
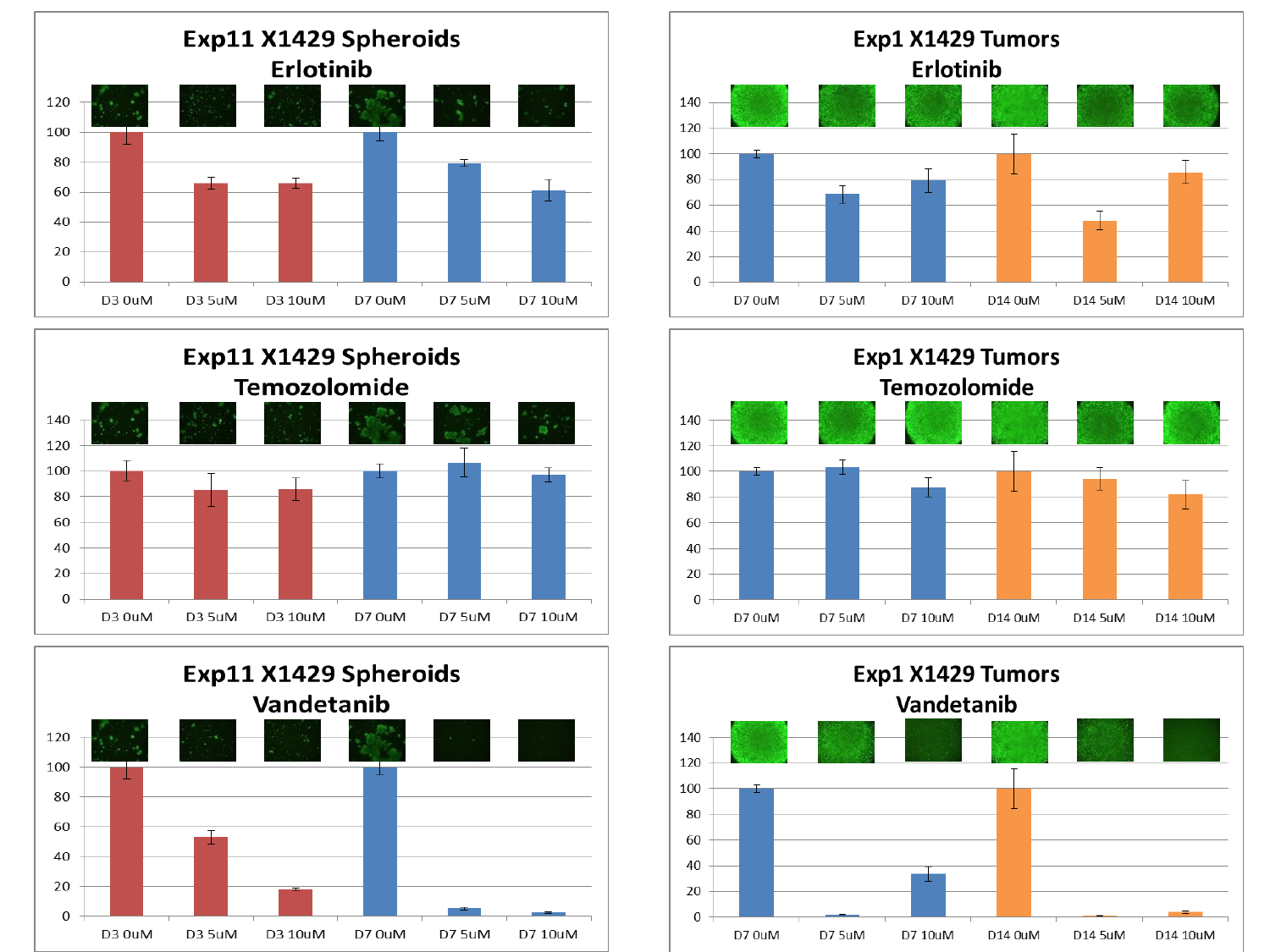

## Slide 4
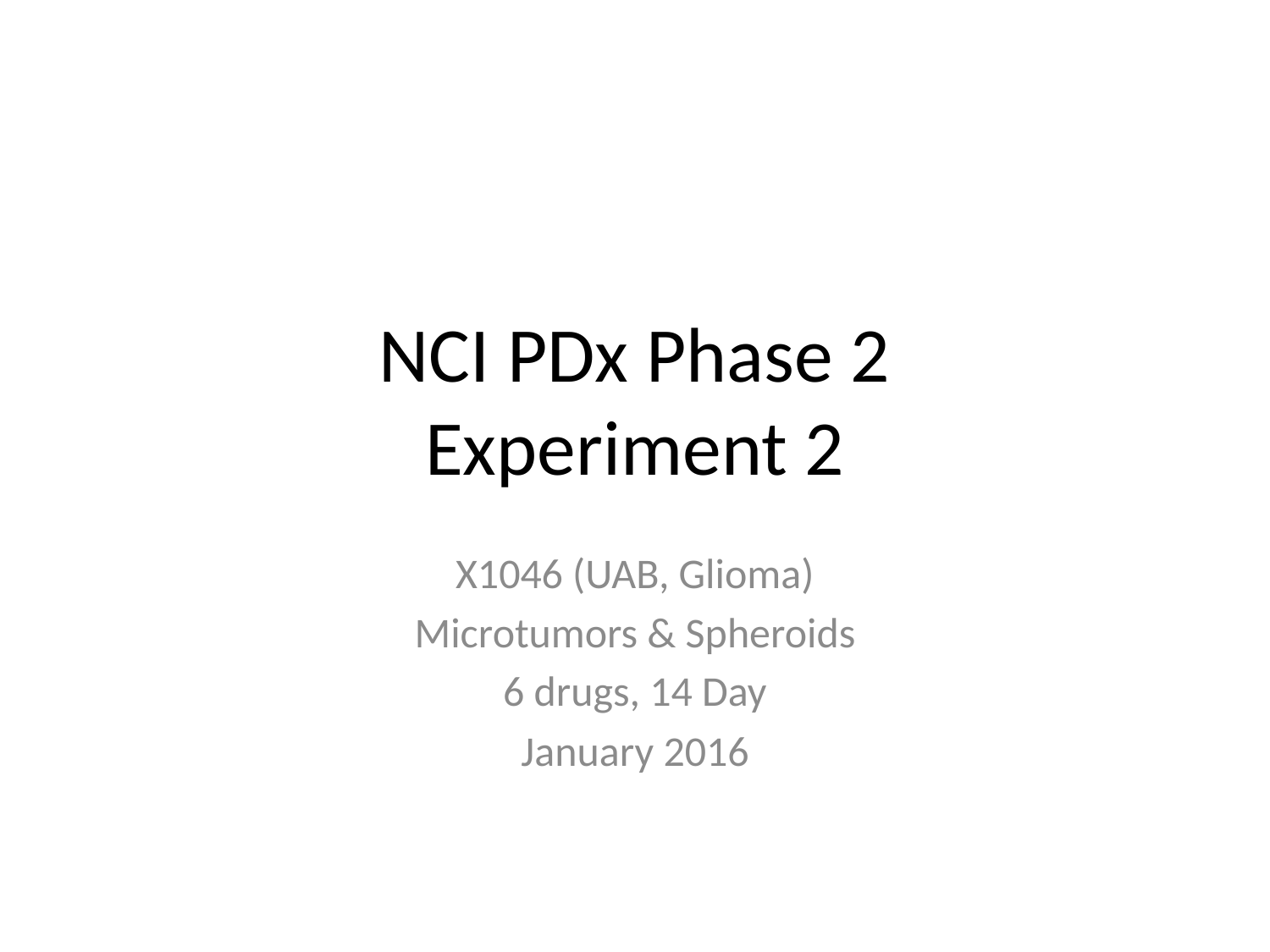

# NCI PDx Phase 2 Experiment 2
X1046 (UAB, Glioma)
Microtumors & Spheroids
6 drugs, 14 Day
January 2016

## Slide 5
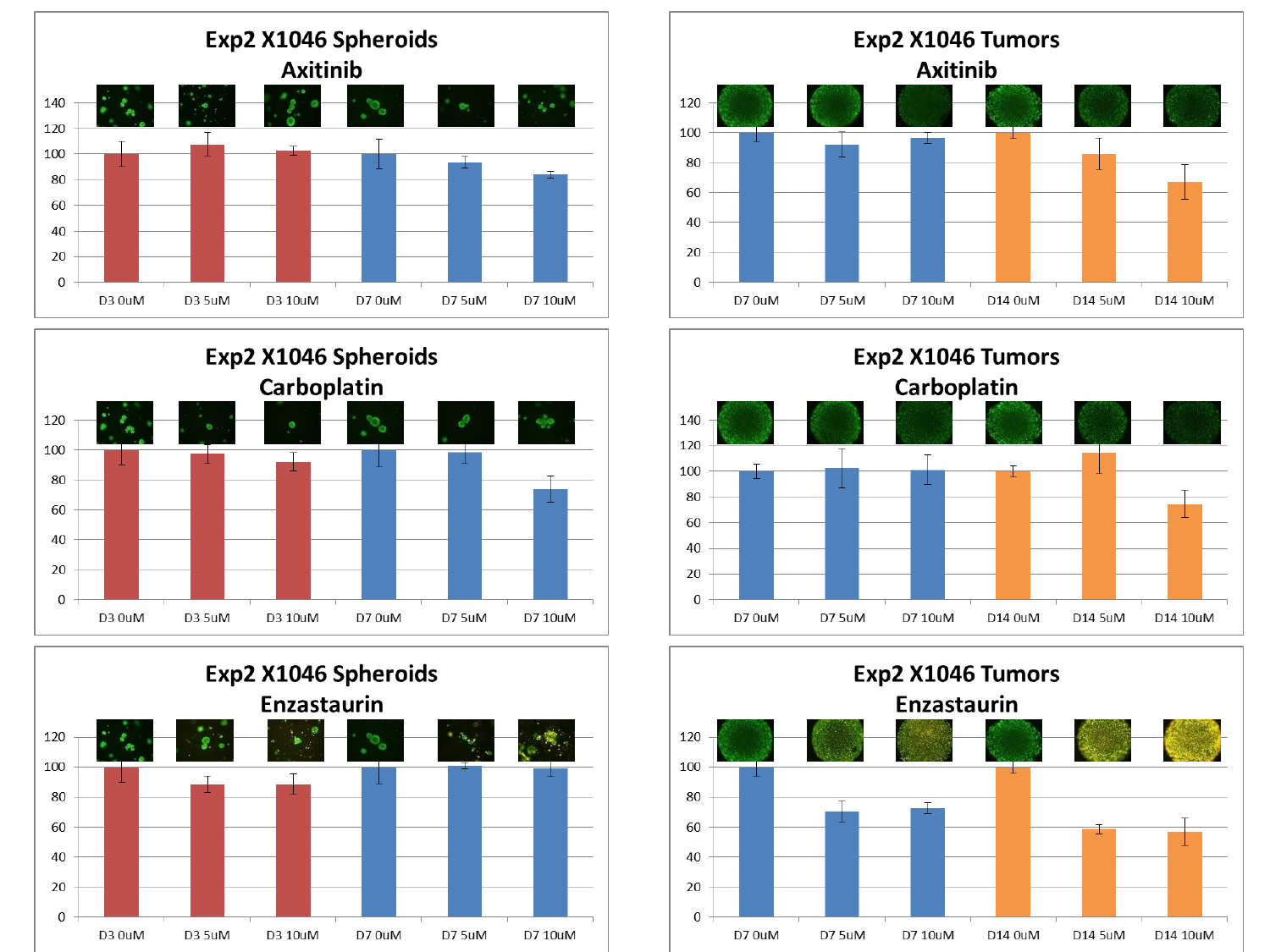

## Slide 6
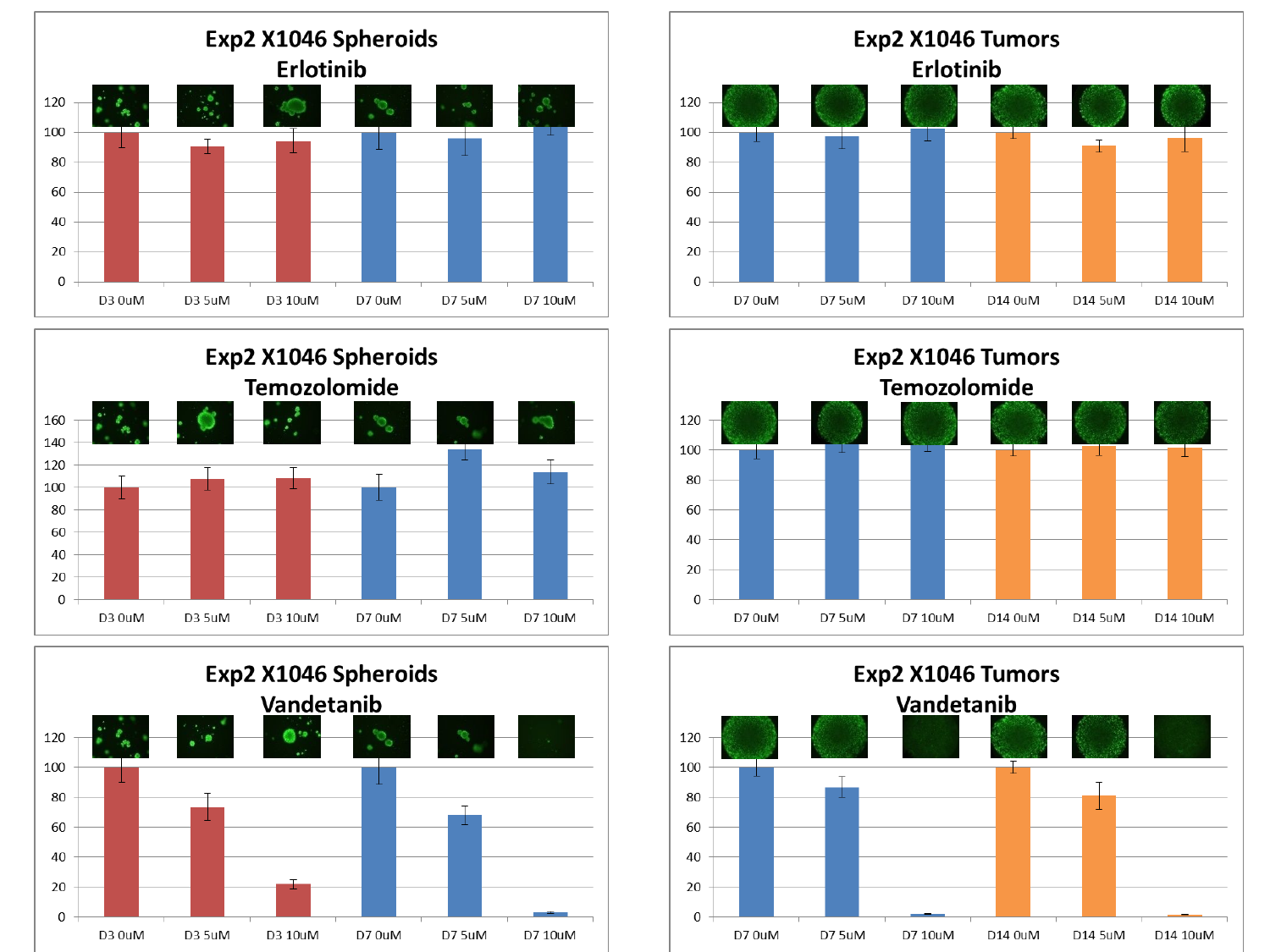

## Slide 7
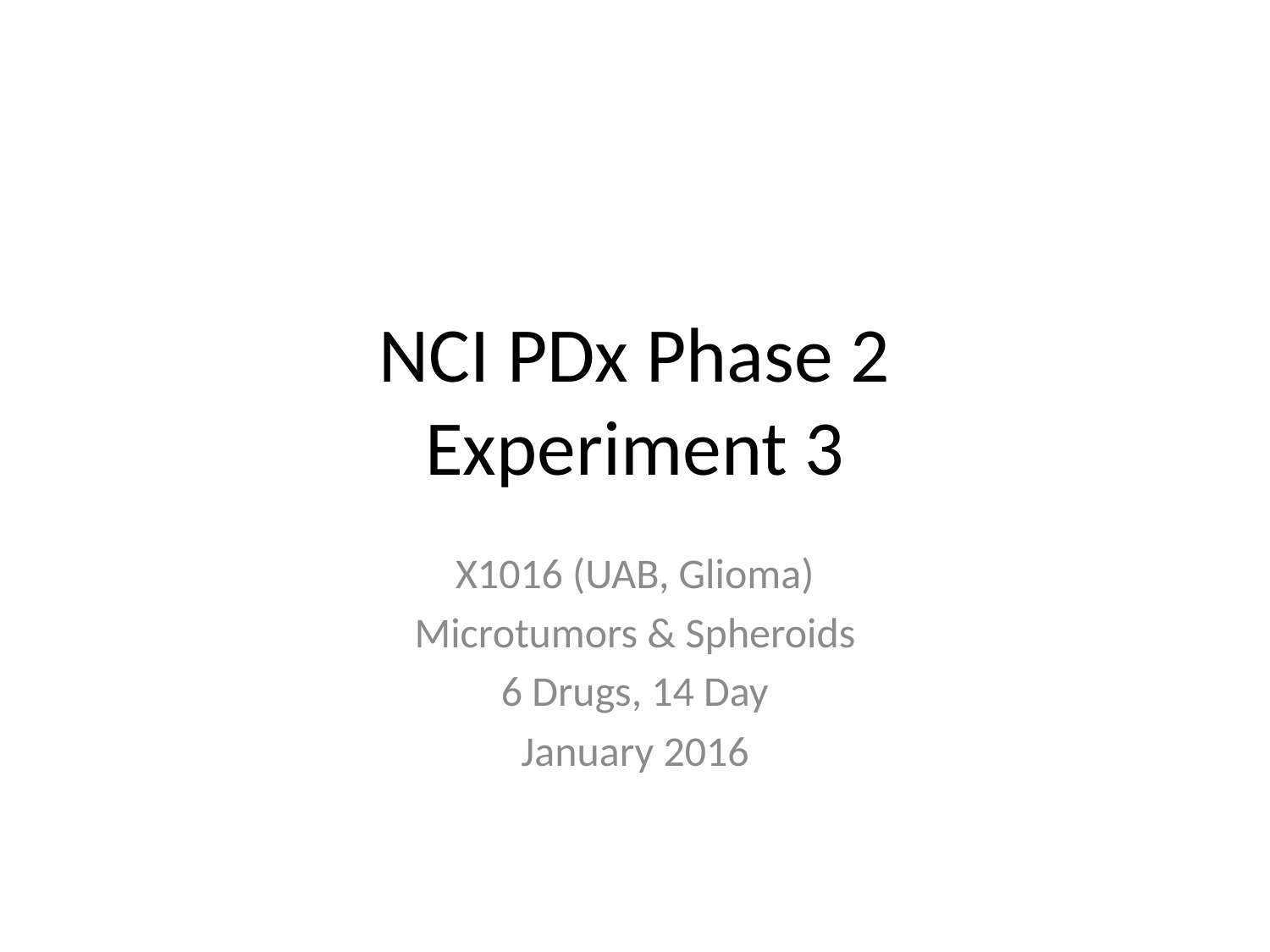

# NCI PDx Phase 2 Experiment 3
X1016 (UAB, Glioma)
Microtumors & Spheroids
6 Drugs, 14 Day
January 2016

## Slide 8
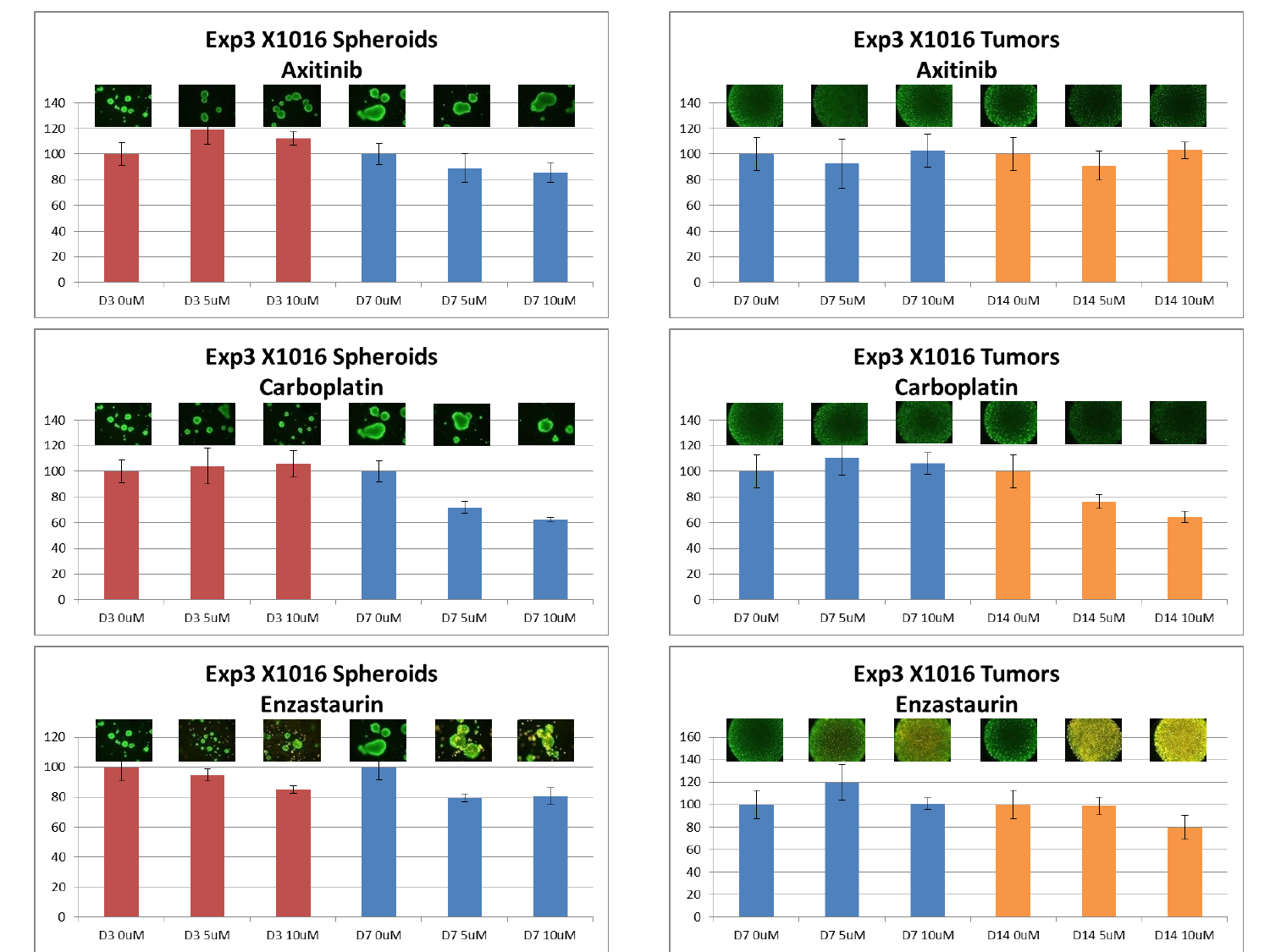

## Slide 9
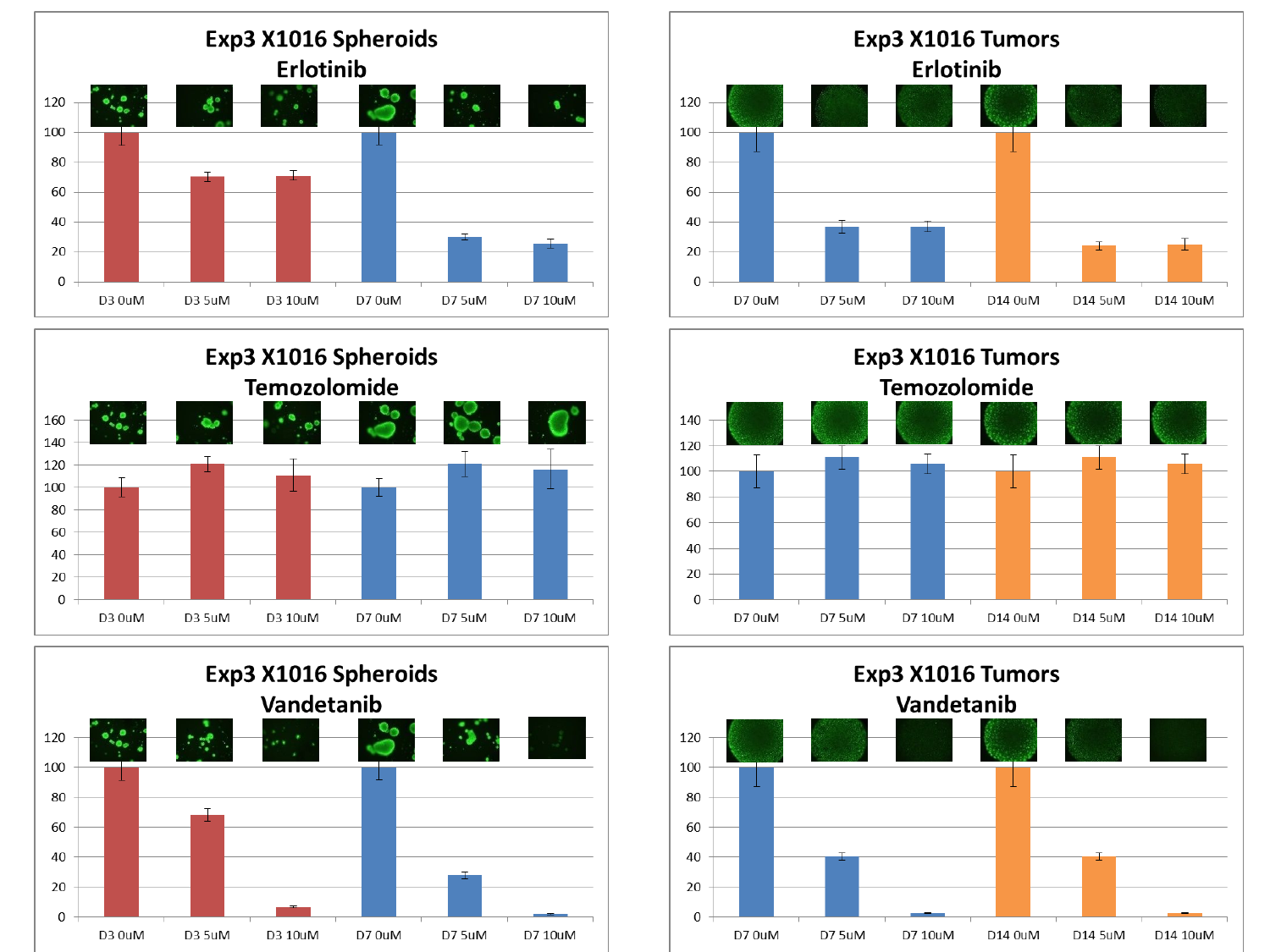

## Slide 10
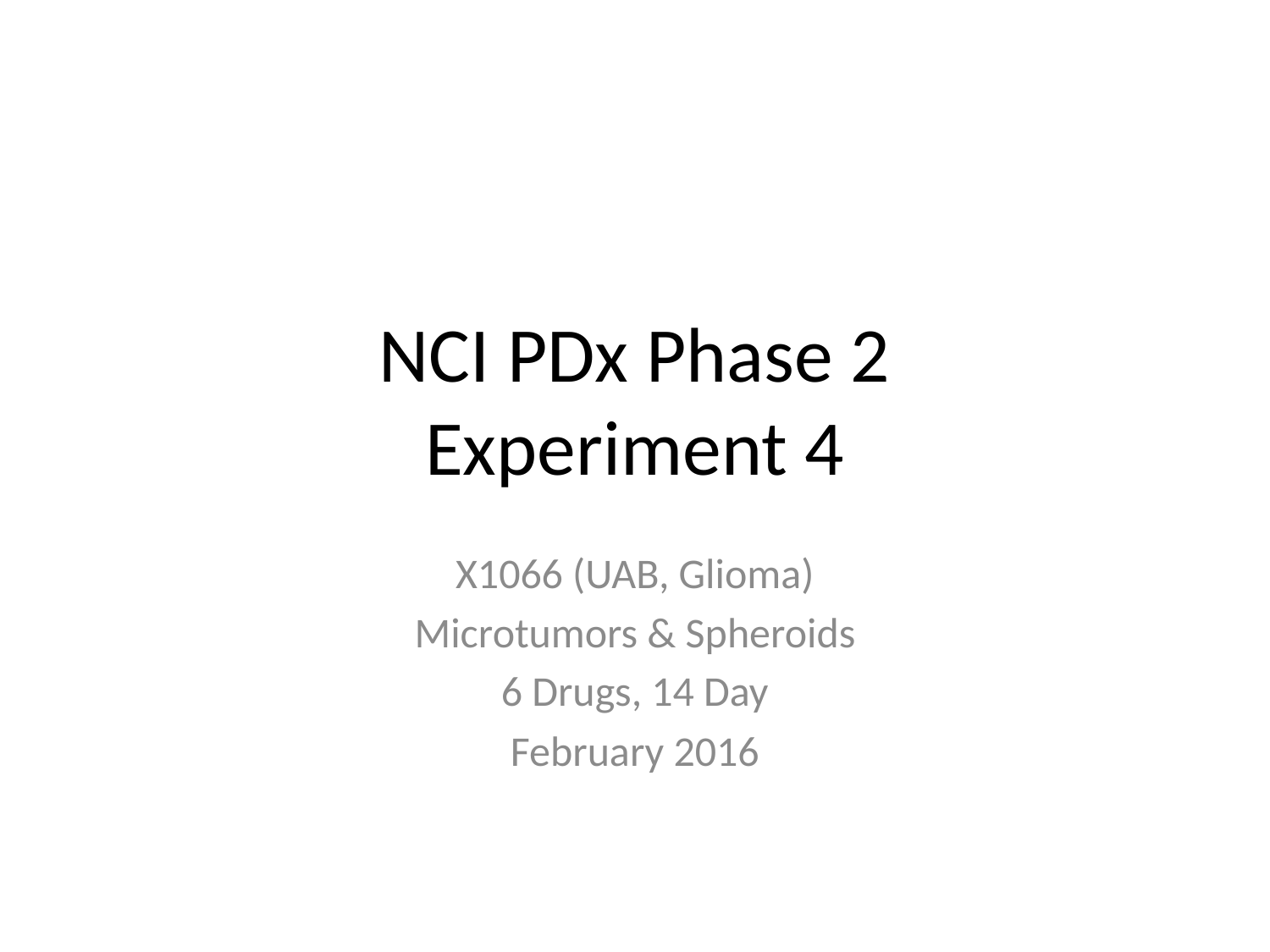

# NCI PDx Phase 2 Experiment 4
X1066 (UAB, Glioma)
Microtumors & Spheroids
6 Drugs, 14 Day
February 2016

## Slide 11
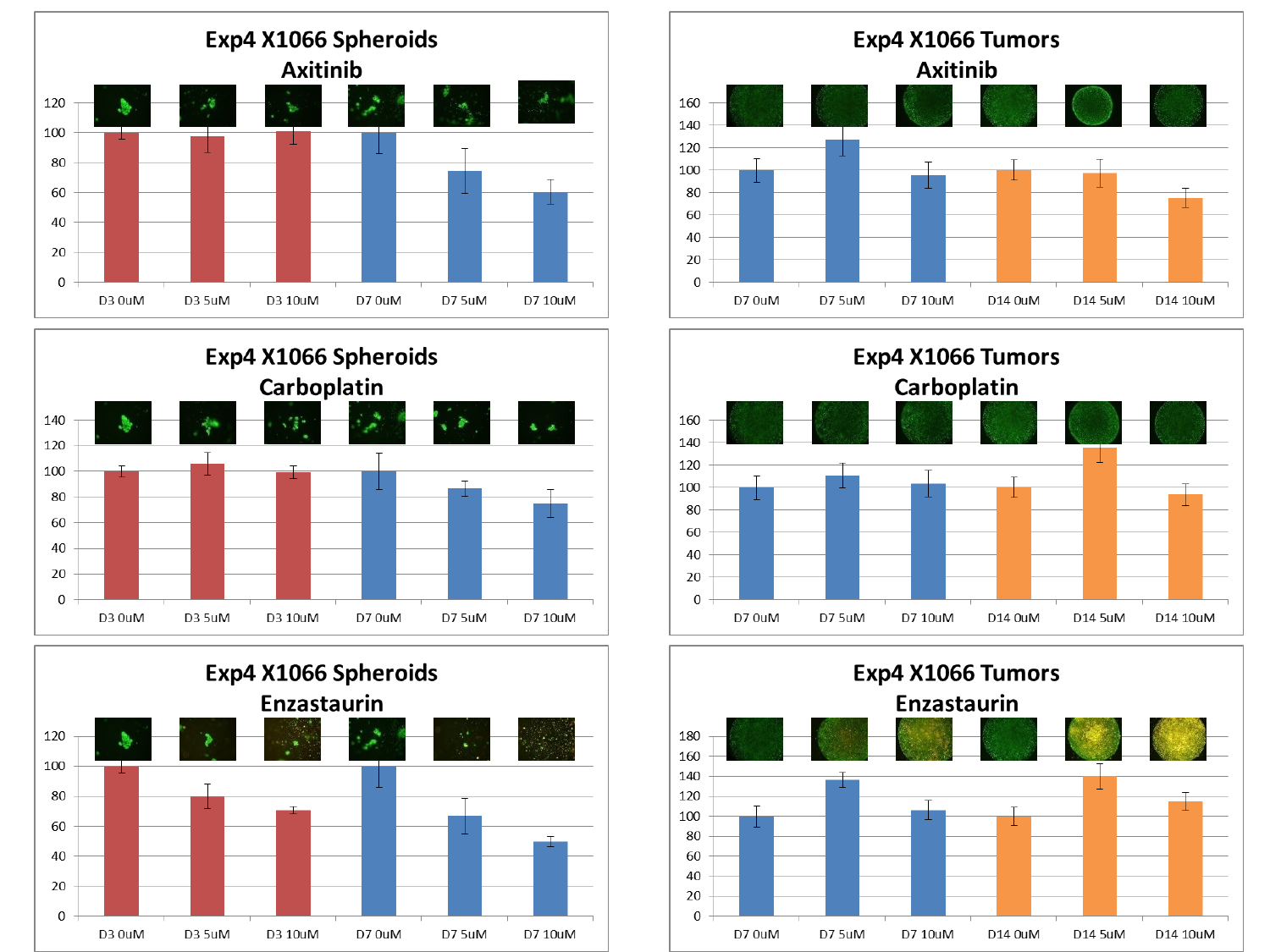

## Slide 12
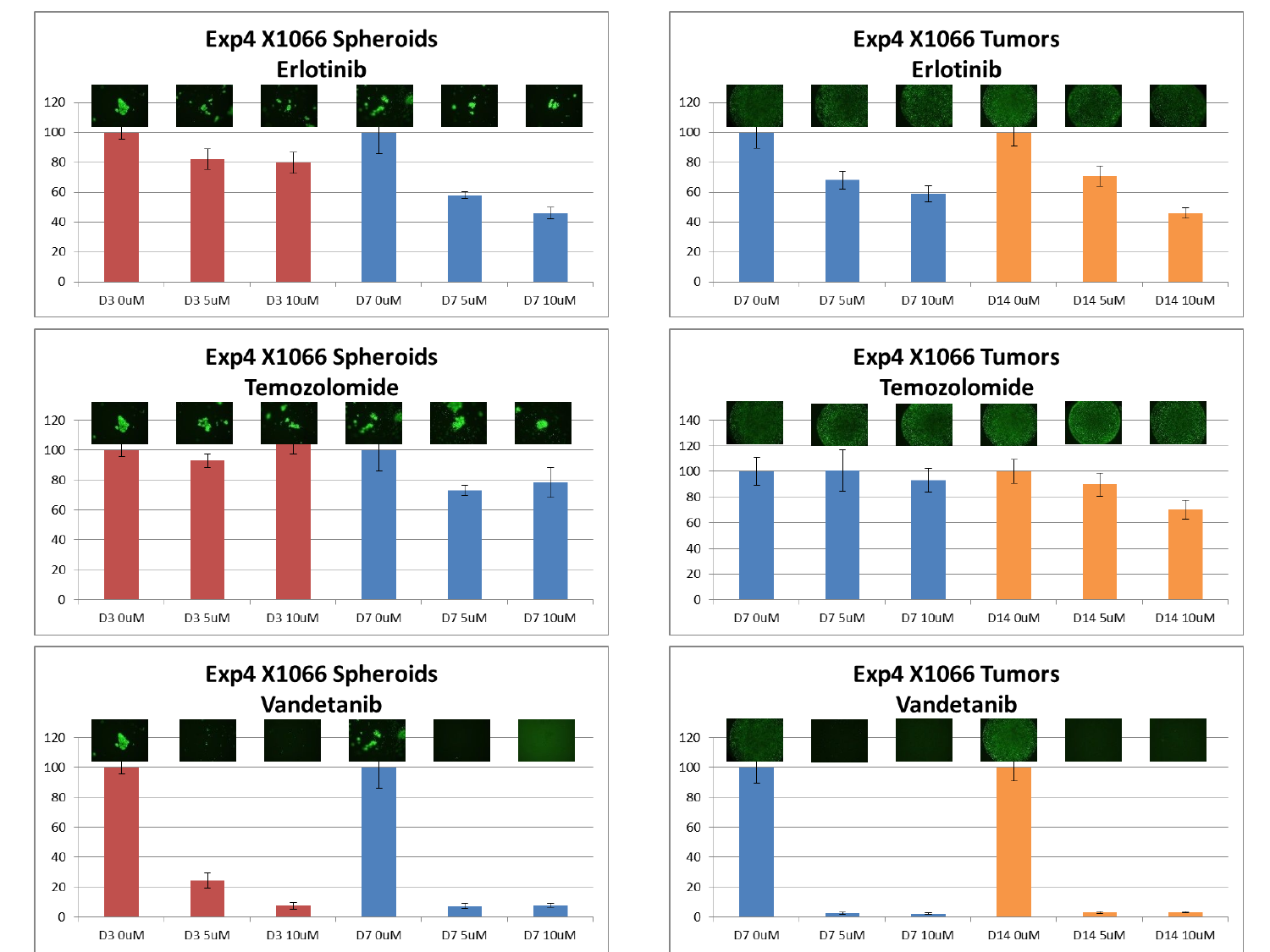

## Slide 13
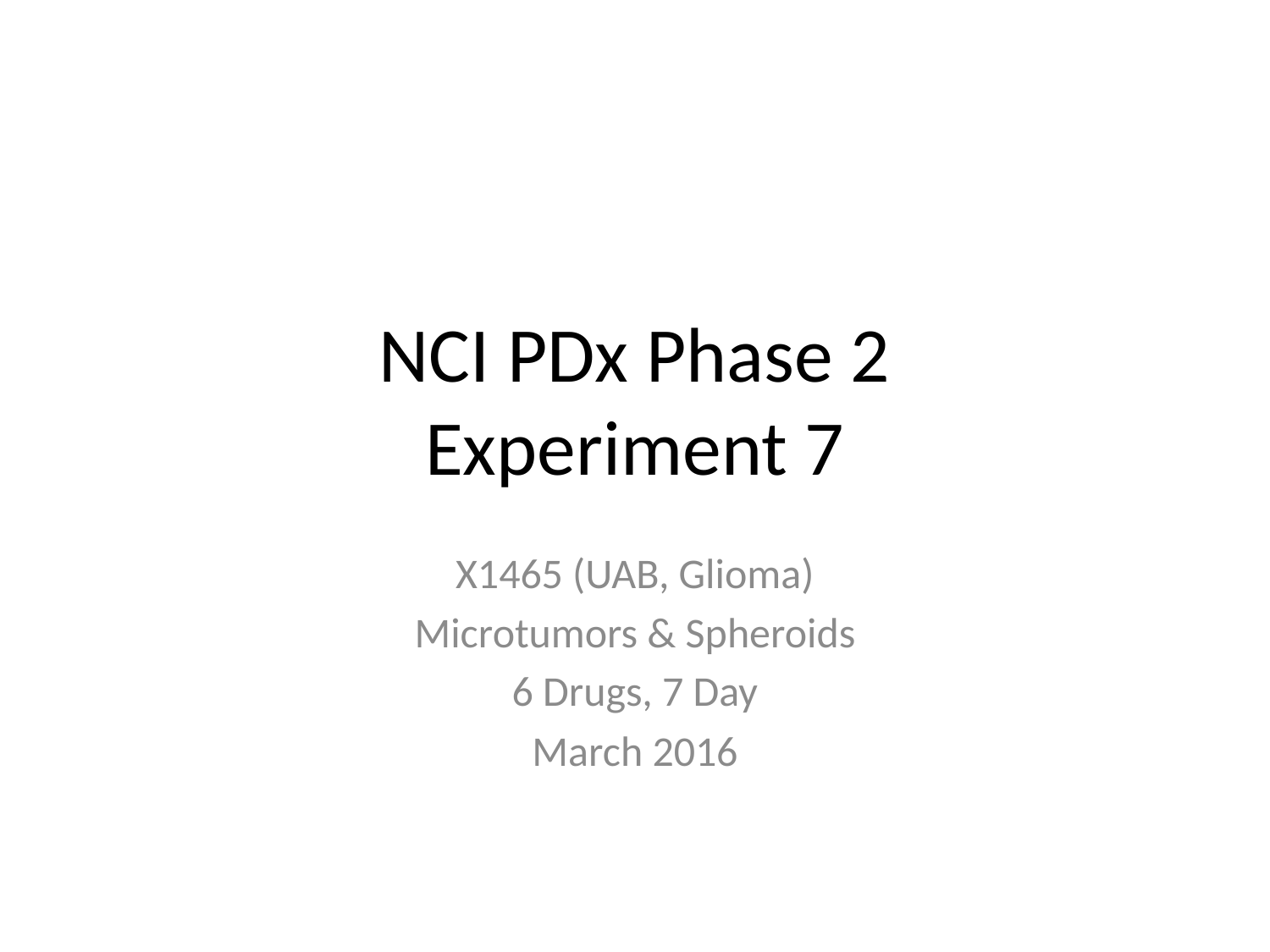

# NCI PDx Phase 2 Experiment 7
X1465 (UAB, Glioma)
Microtumors & Spheroids
6 Drugs, 7 Day
March 2016

## Slide 14
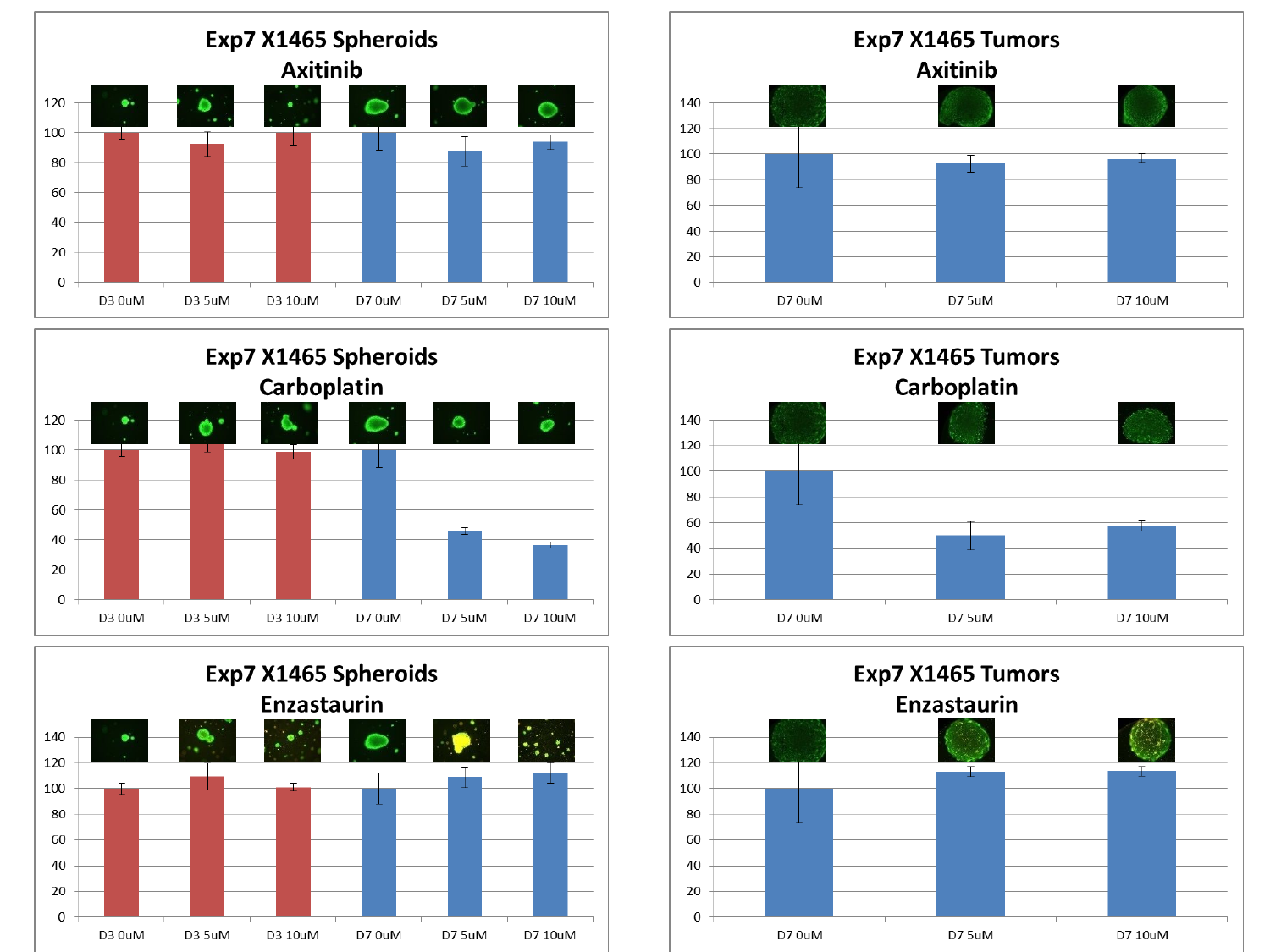

## Slide 15
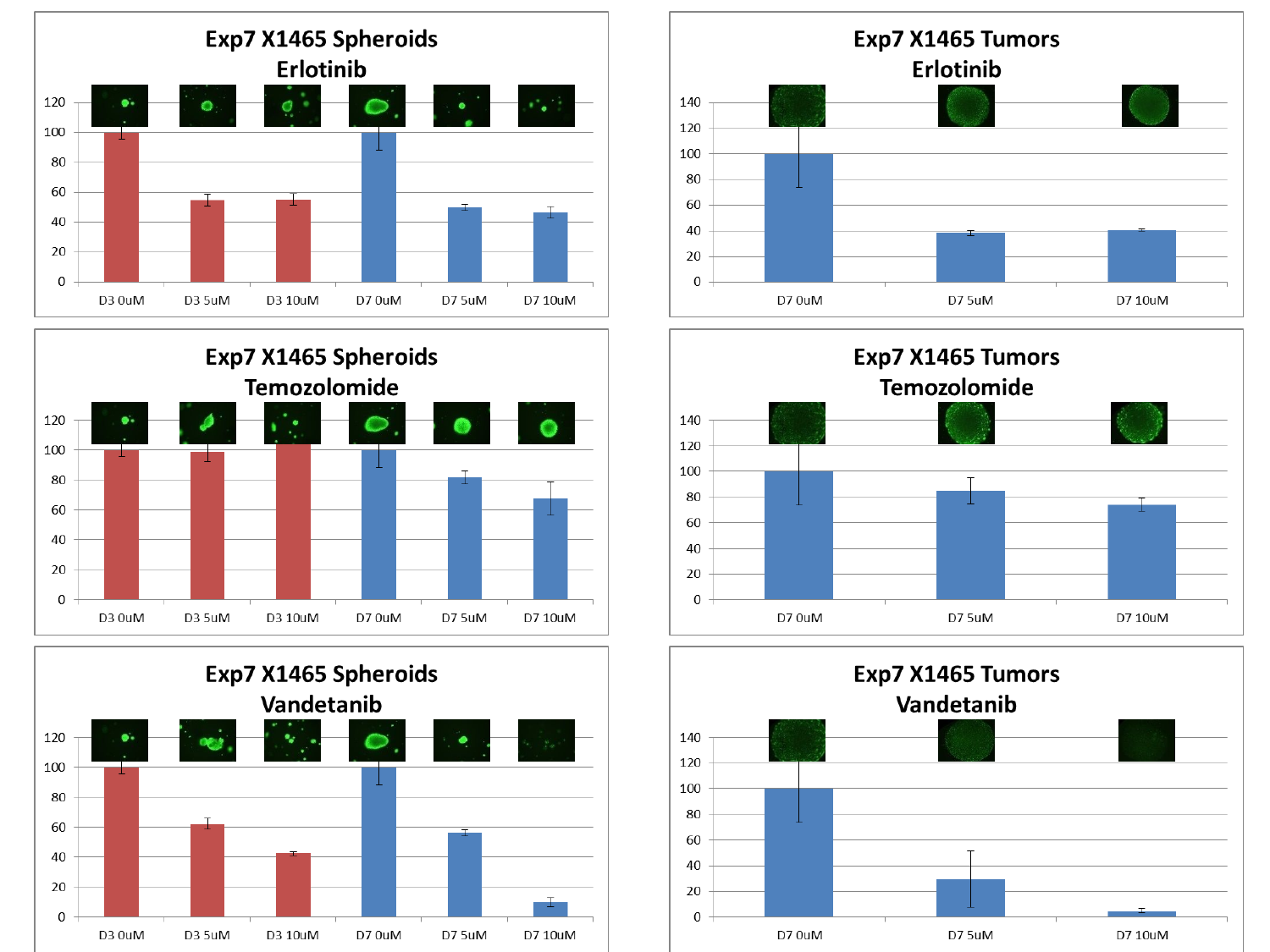

## Slide 16
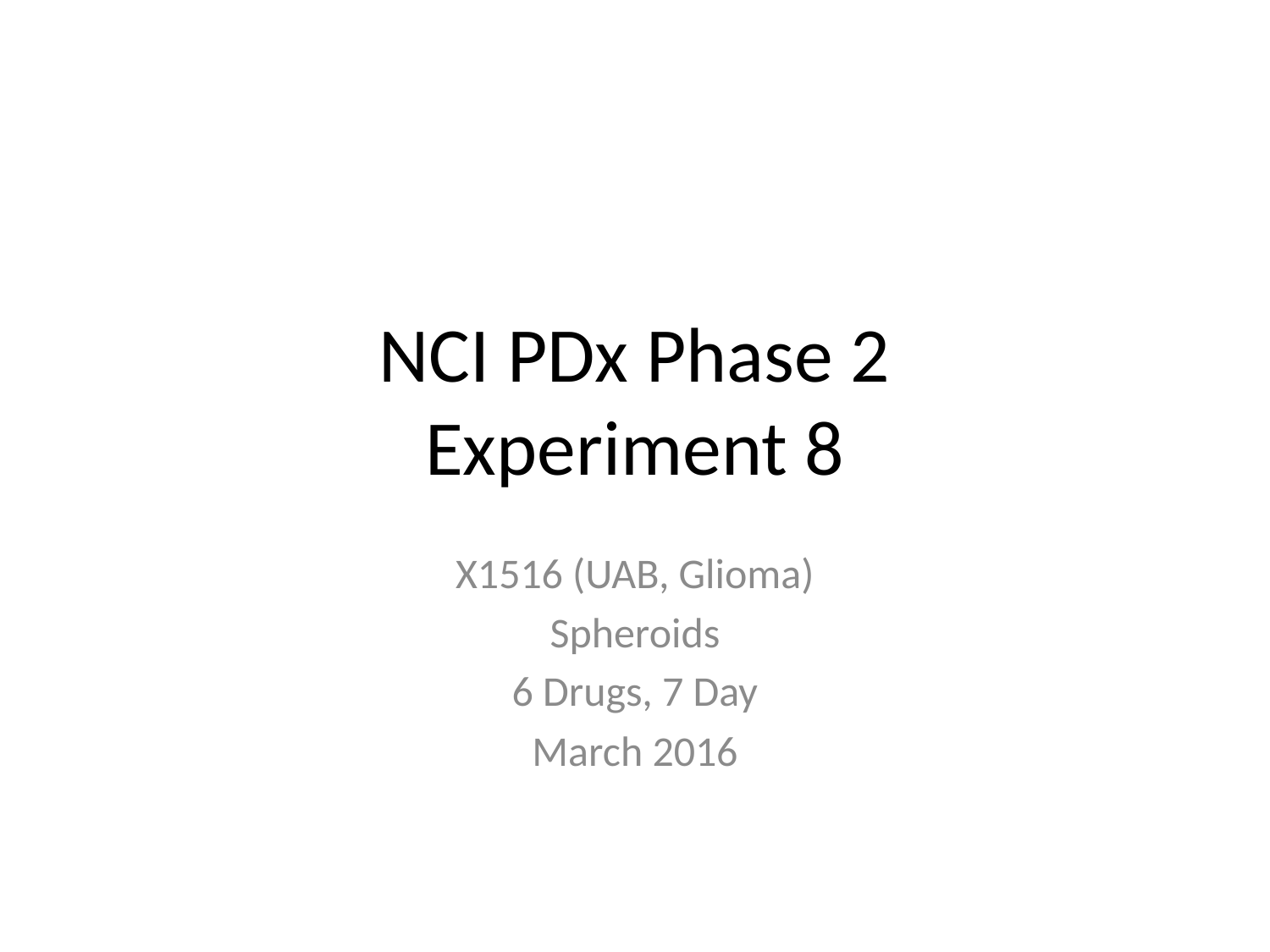

# NCI PDx Phase 2 Experiment 8
X1516 (UAB, Glioma)
Spheroids
6 Drugs, 7 Day
March 2016

## Slide 17
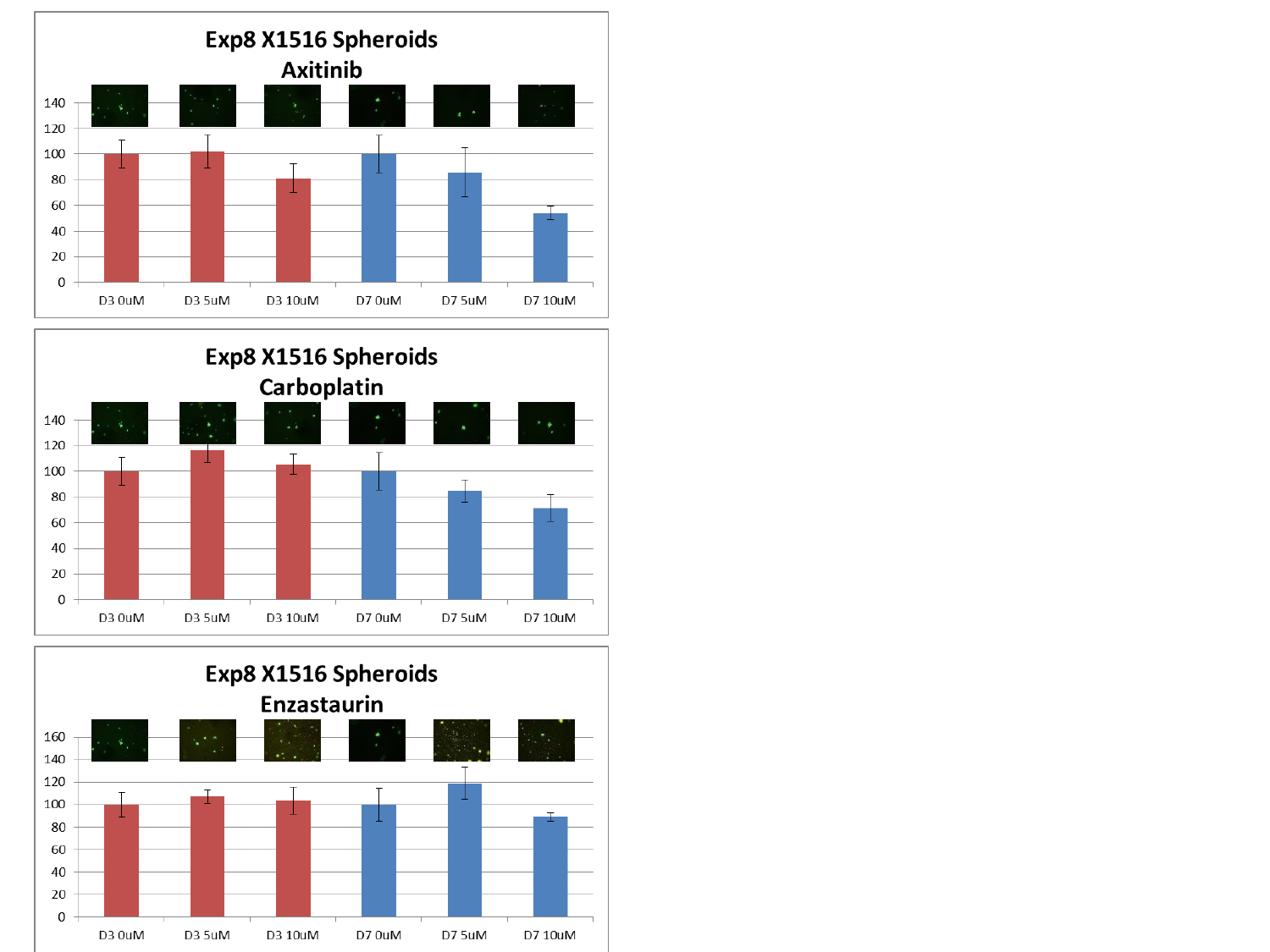

## Slide 18
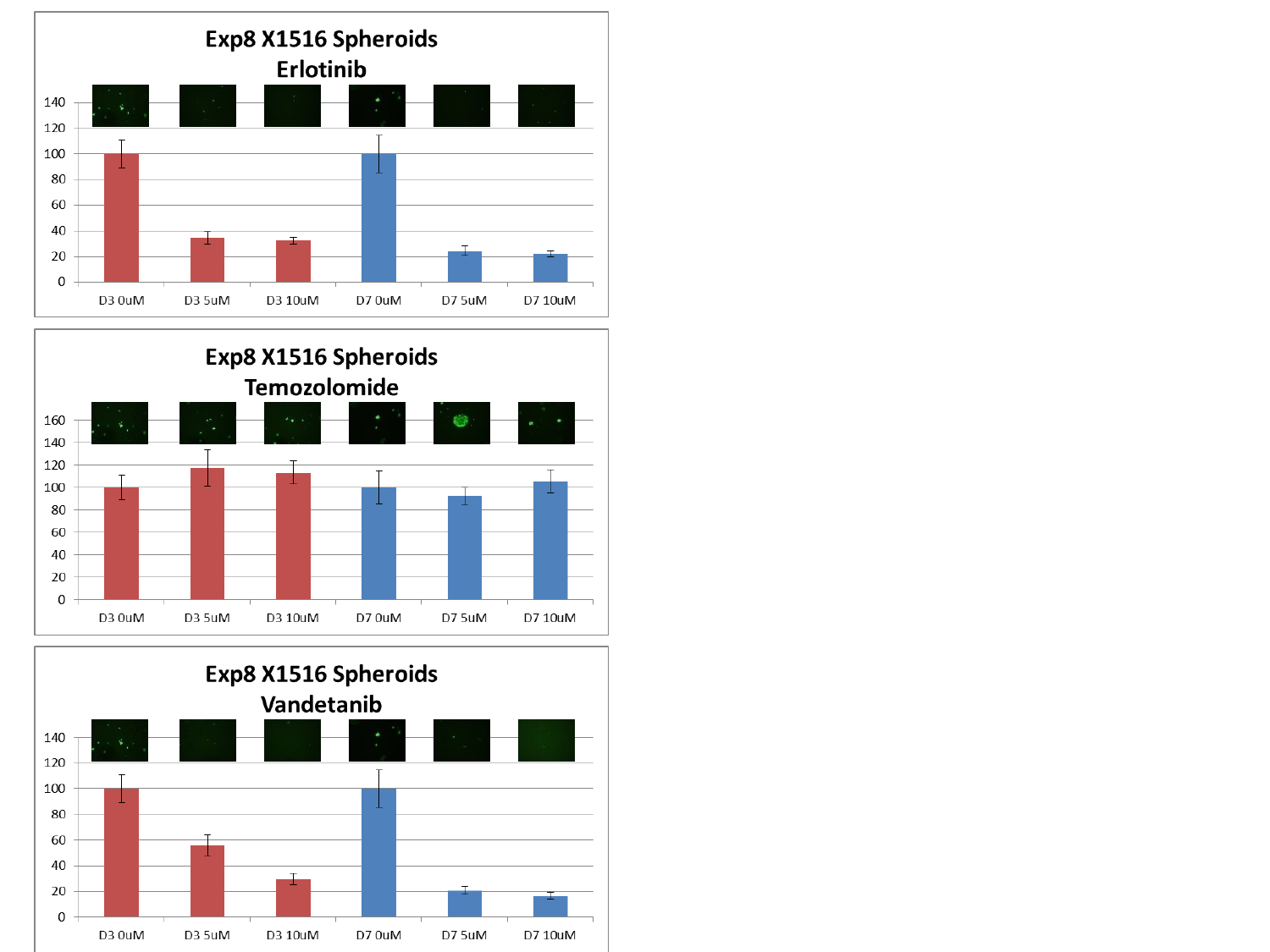

## Slide 19
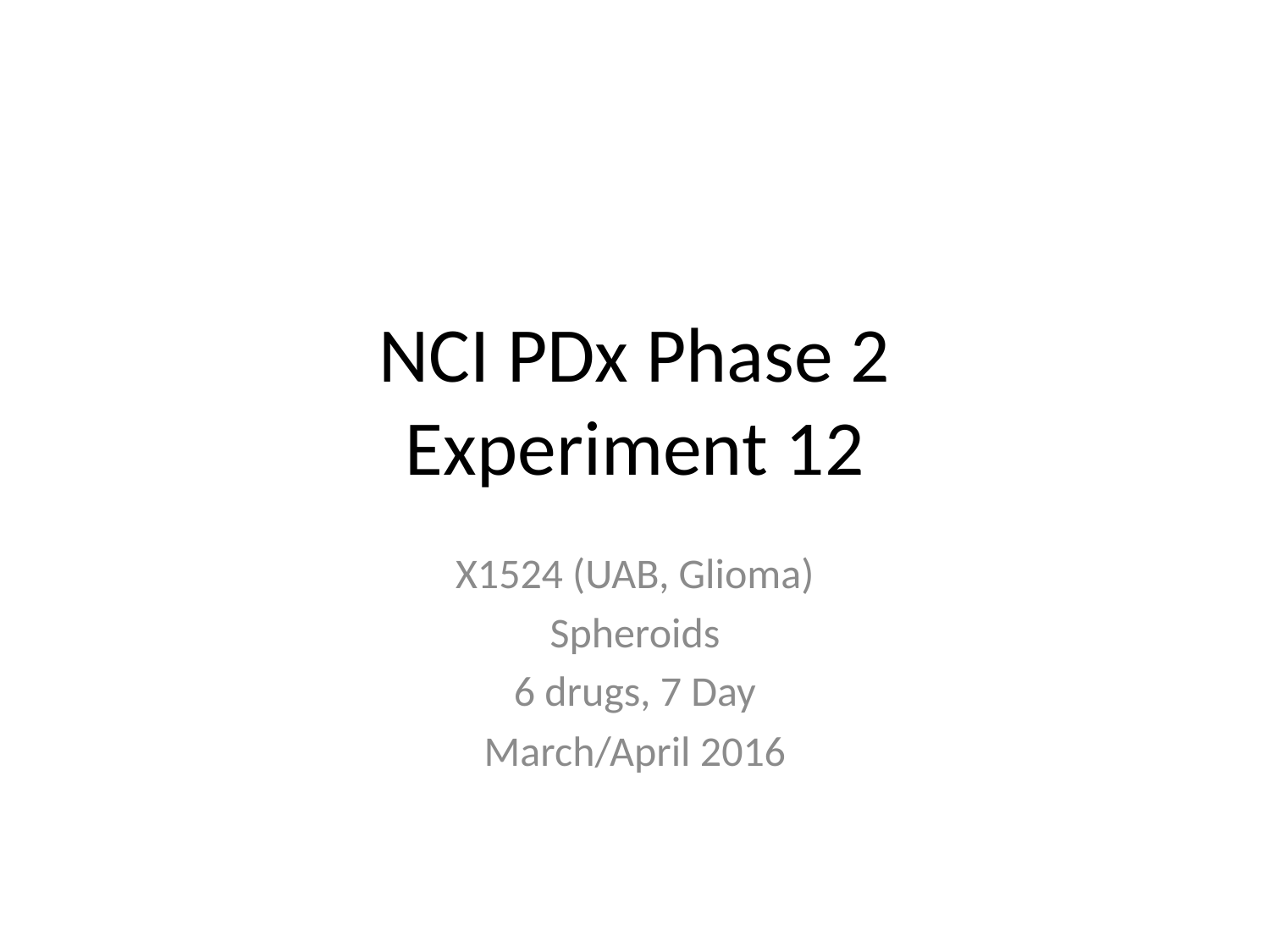

# NCI PDx Phase 2 Experiment 12
X1524 (UAB, Glioma)
Spheroids
6 drugs, 7 Day
March/April 2016

## Slide 20
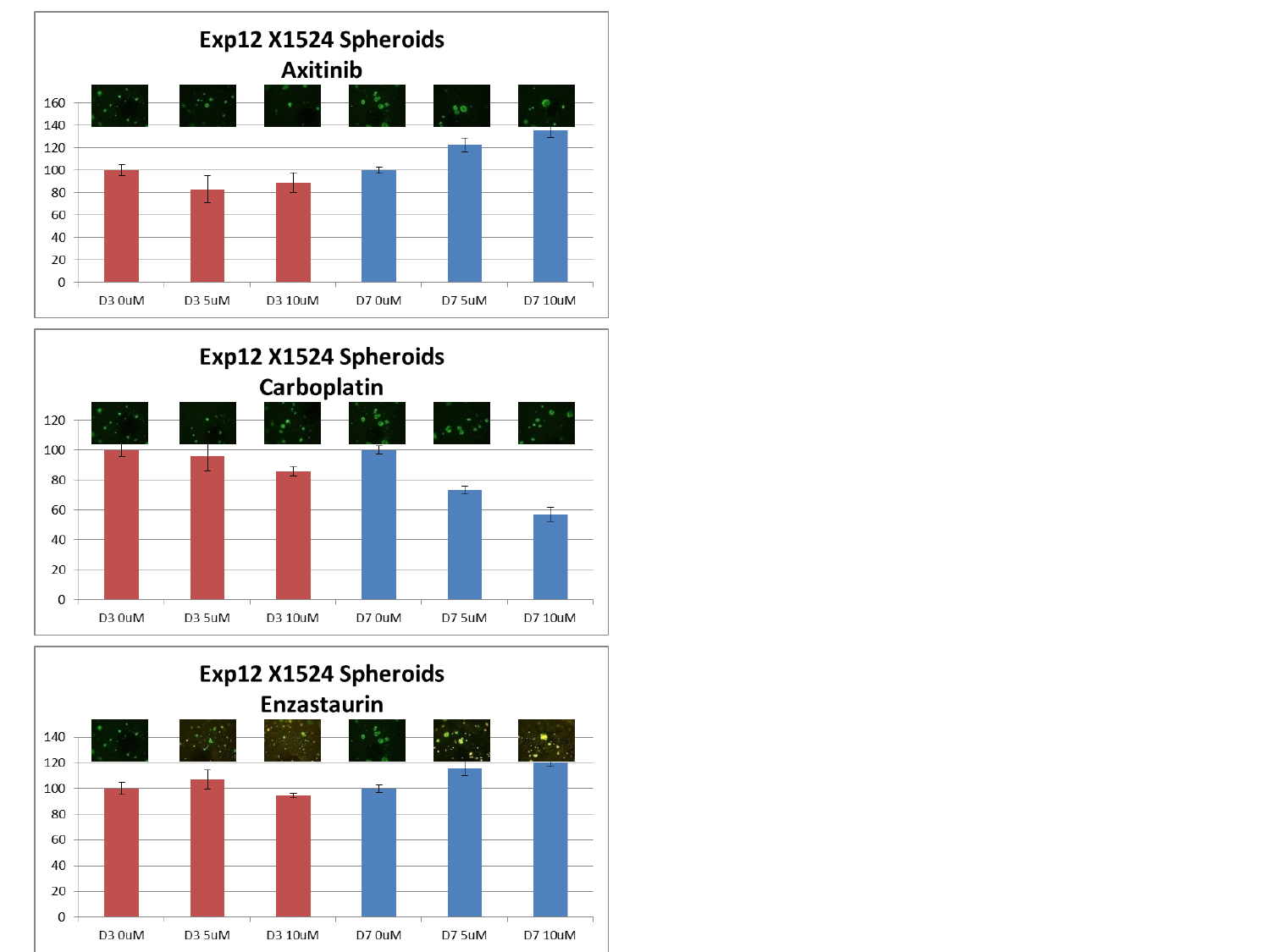

## Slide 21
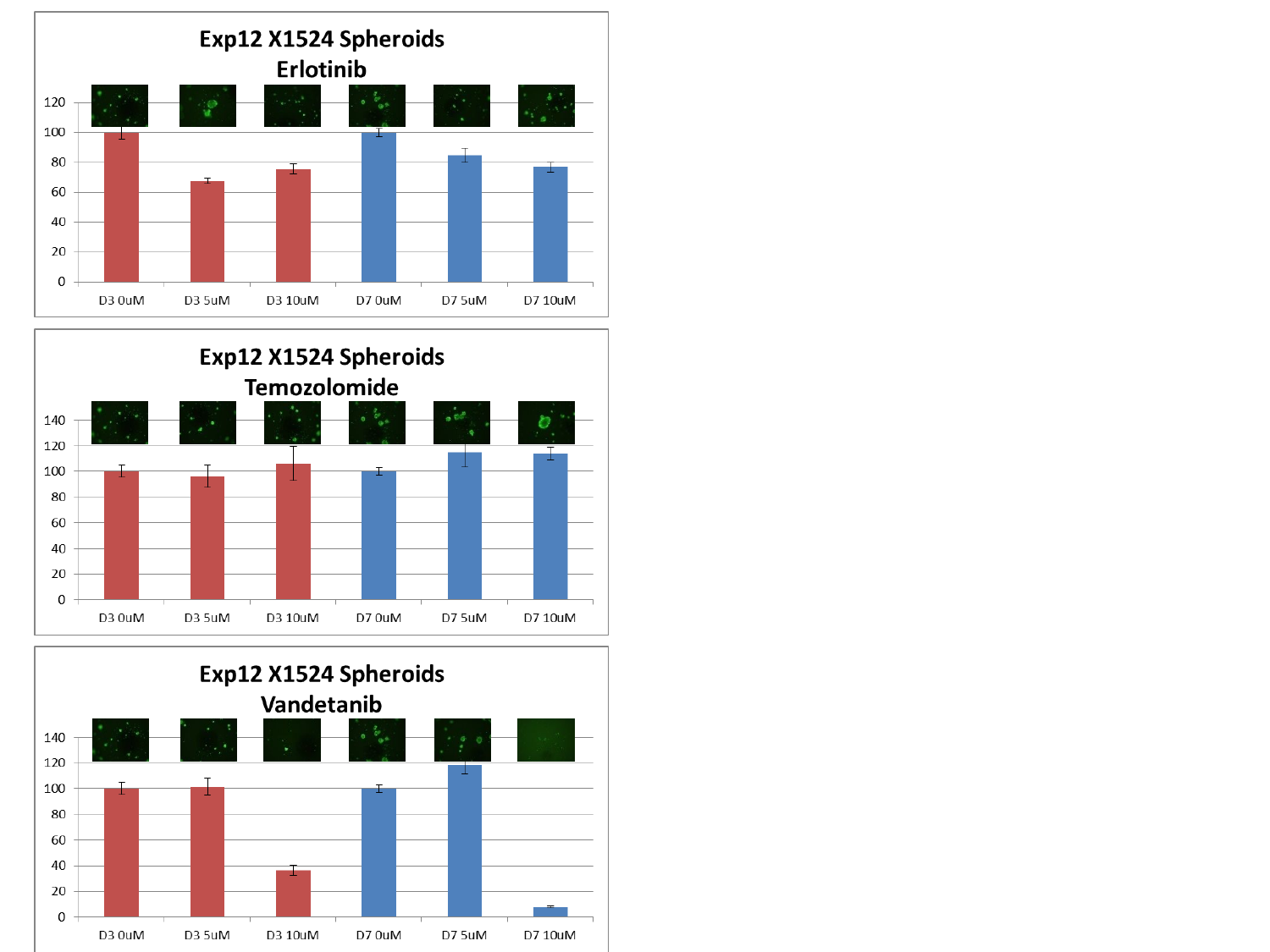

## Slide 22
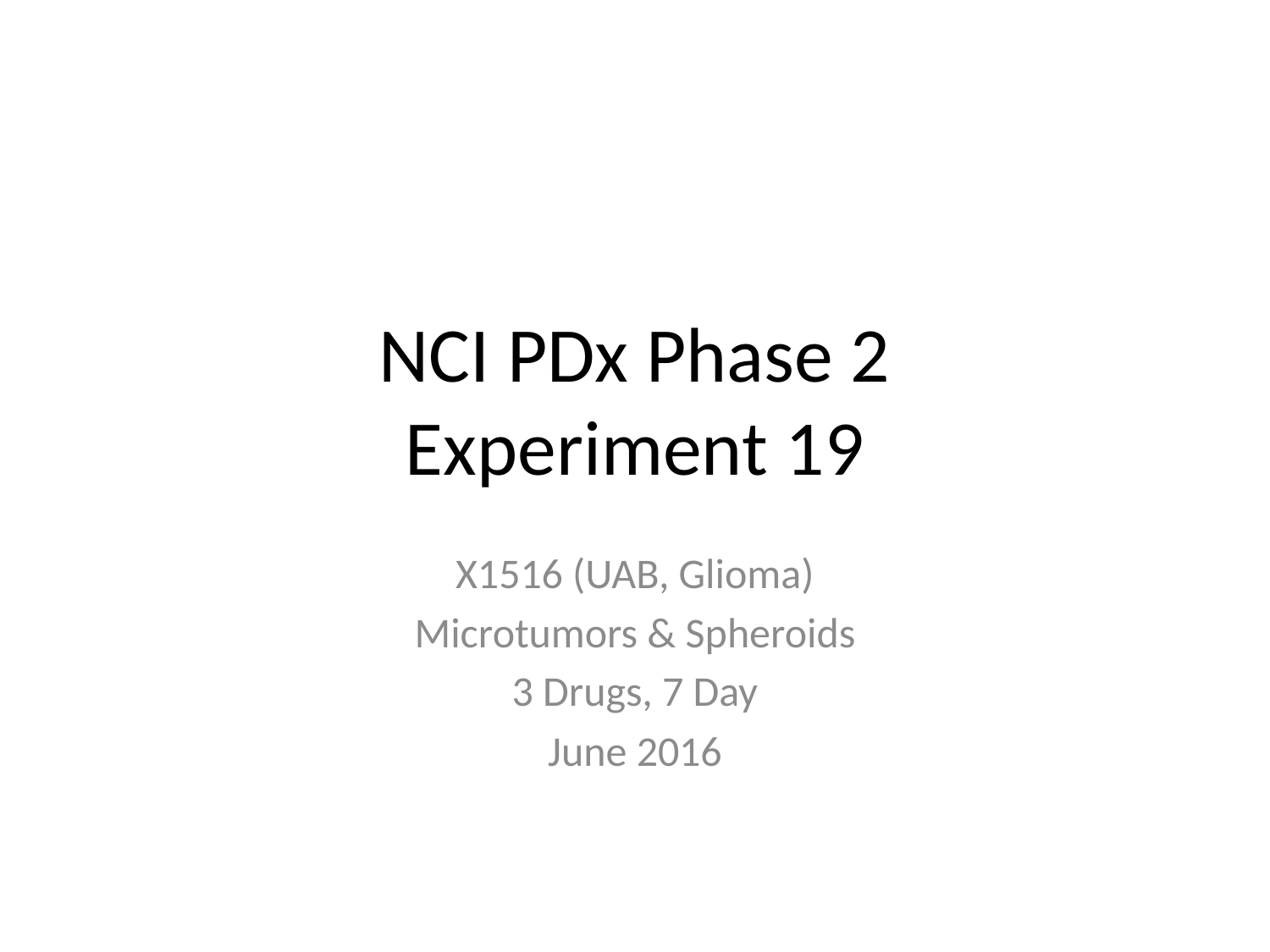

# NCI PDx Phase 2 Experiment 19
X1516 (UAB, Glioma)
Microtumors & Spheroids
3 Drugs, 7 Day
June 2016

## Slide 23
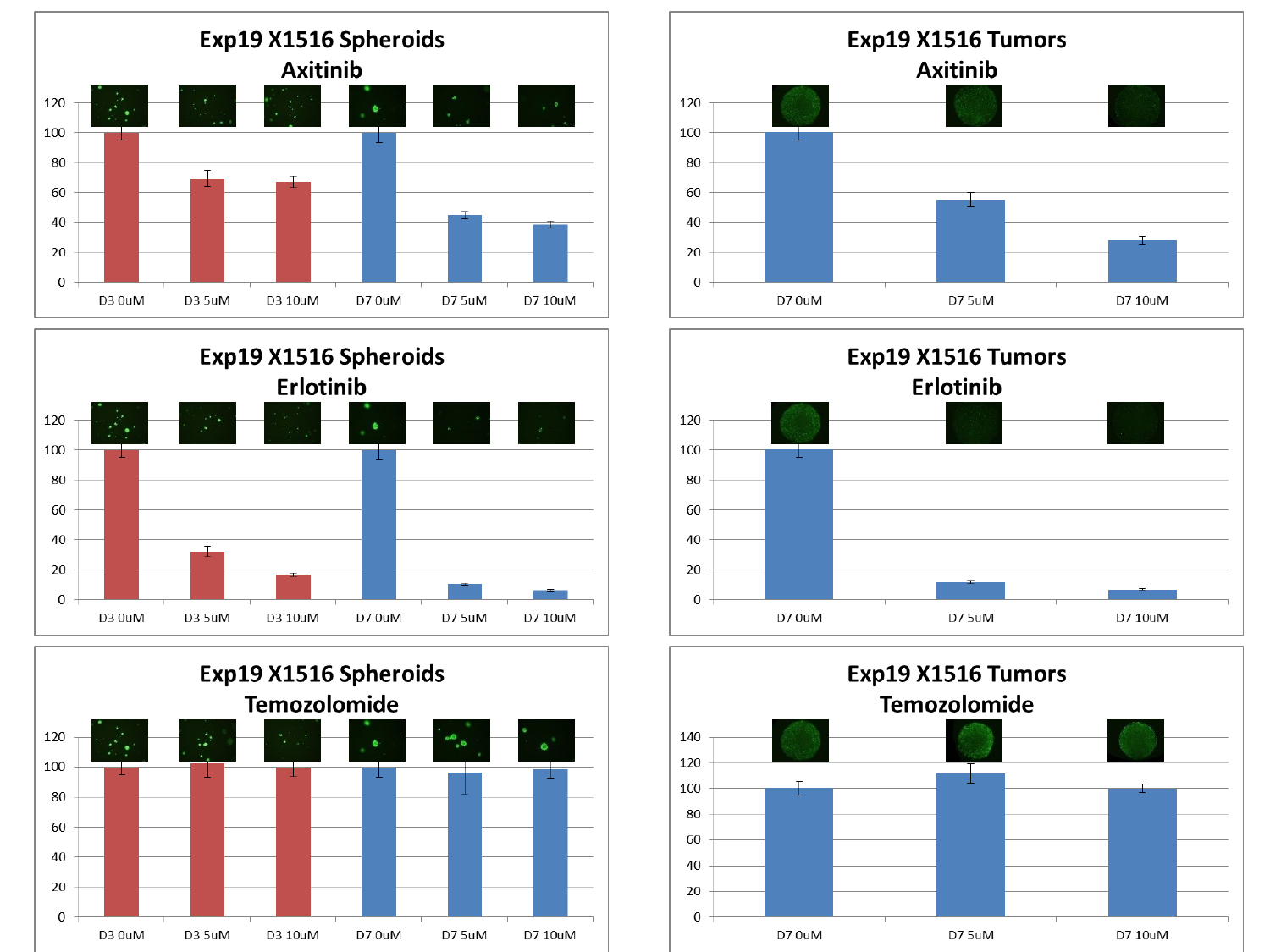

## Slide 24
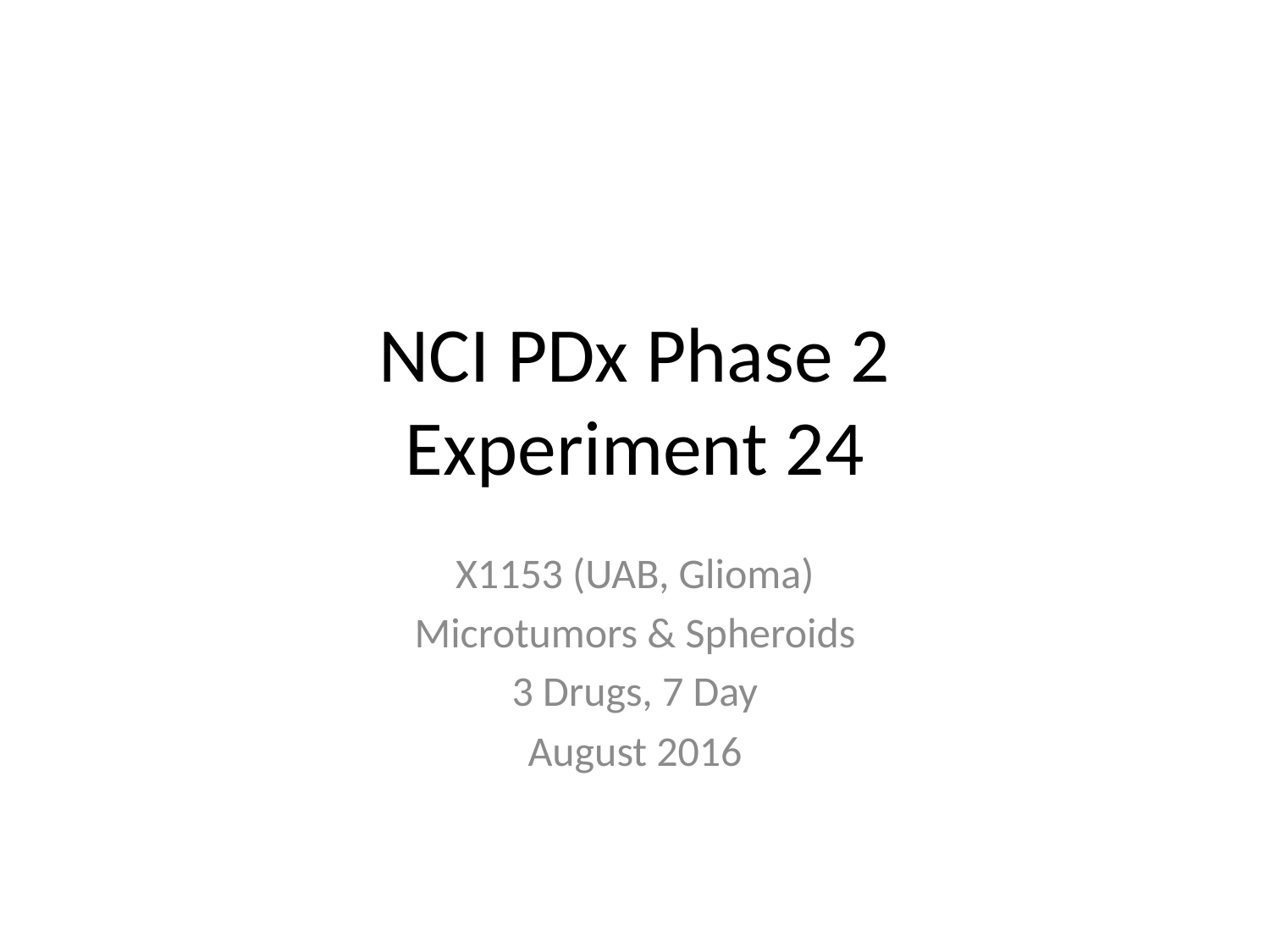

# NCI PDx Phase 2 Experiment 24
X1153 (UAB, Glioma)
Microtumors & Spheroids
3 Drugs, 7 Day
August 2016

## Slide 25
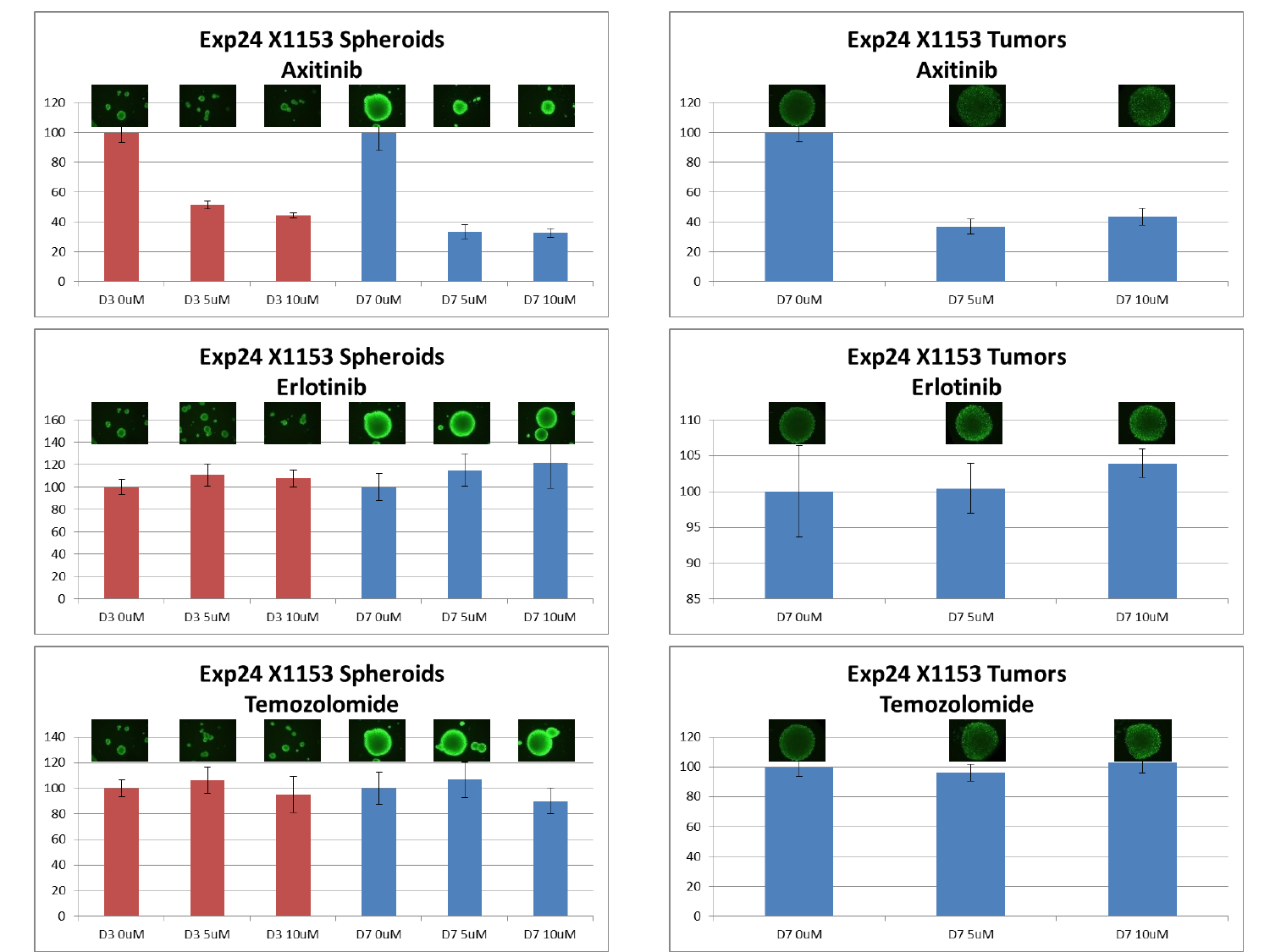

## Slide 26
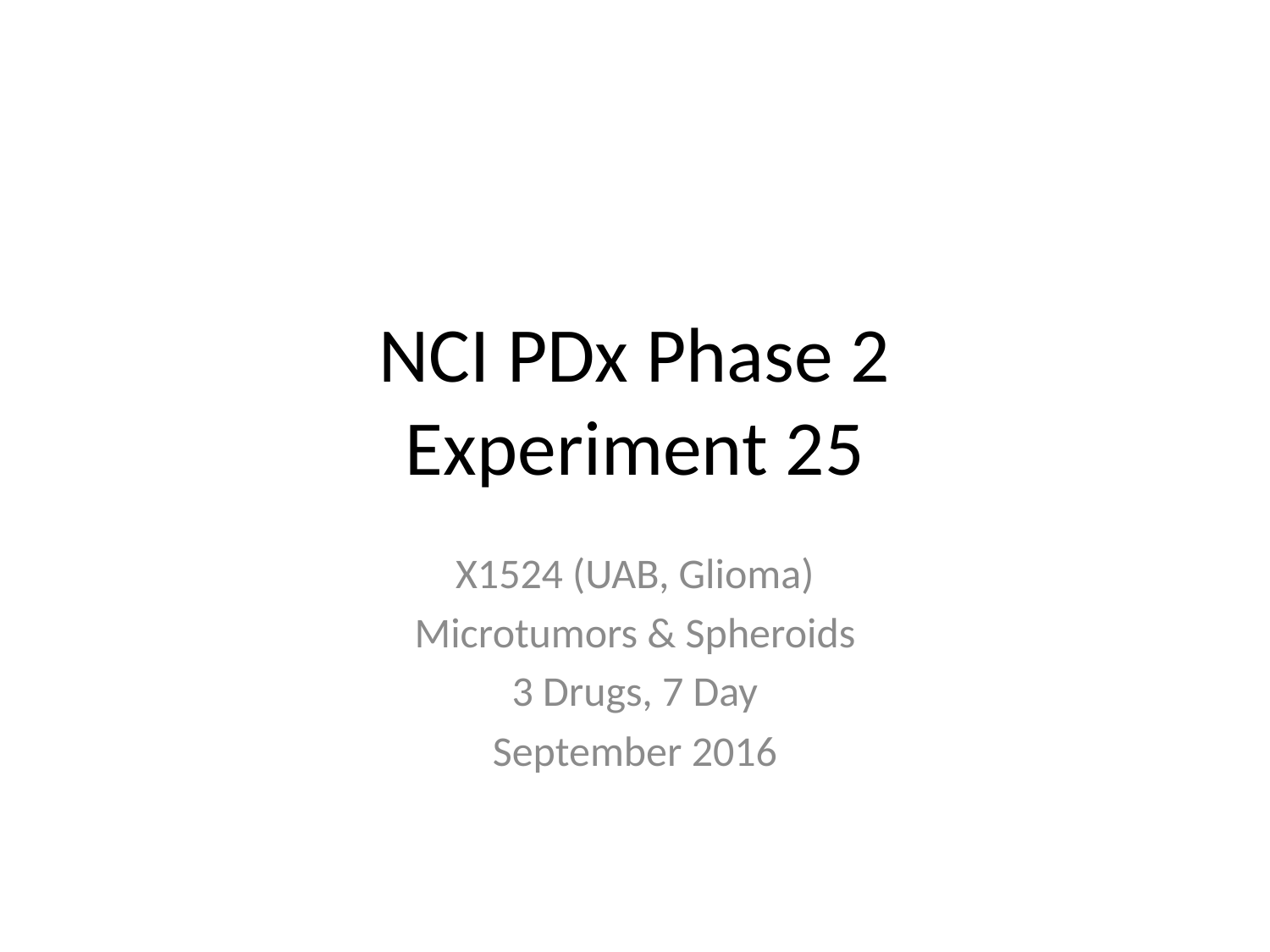

# NCI PDx Phase 2 Experiment 25
X1524 (UAB, Glioma)
Microtumors & Spheroids
3 Drugs, 7 Day
September 2016

## Slide 27
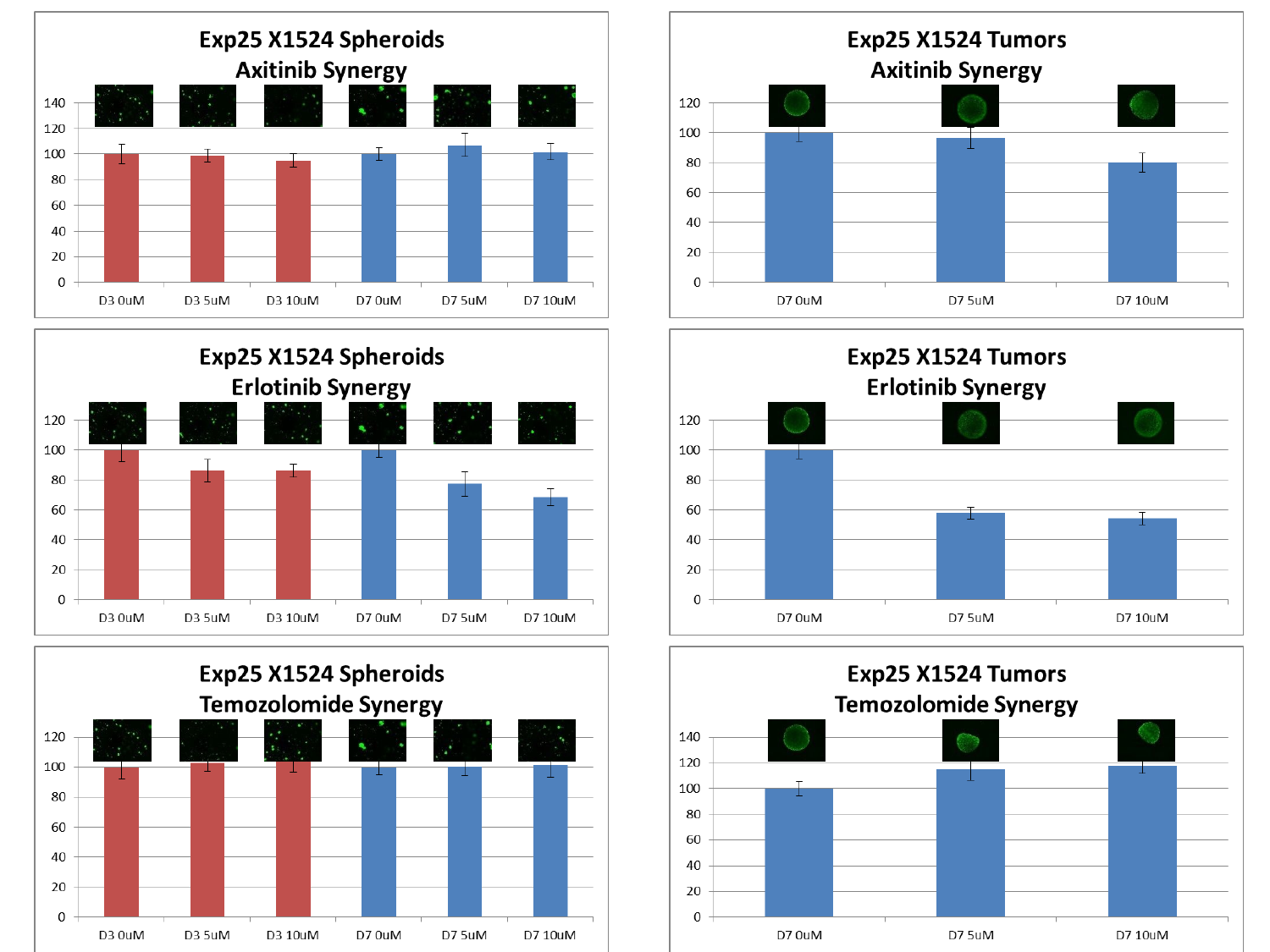

## Slide 28
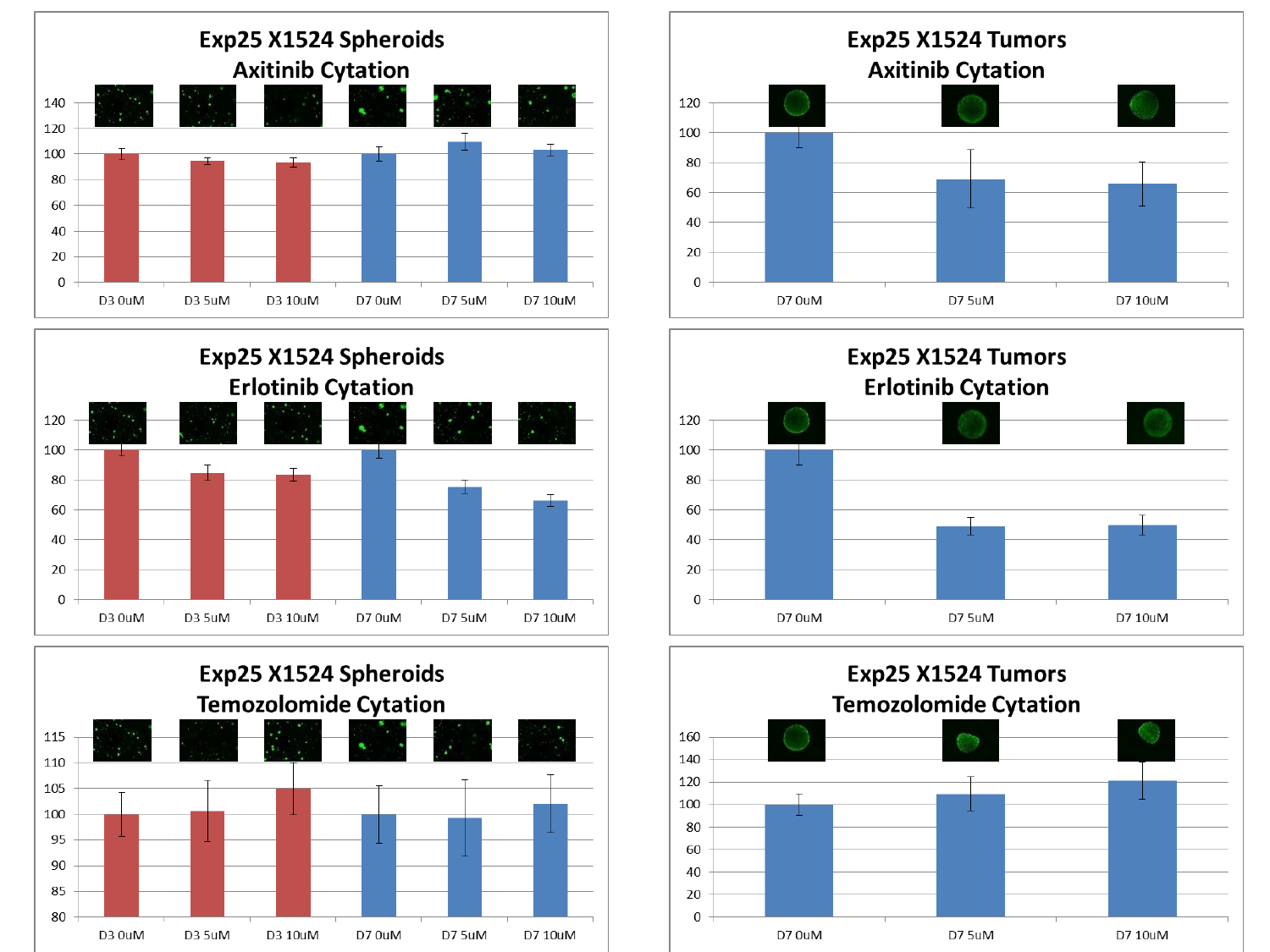

## Slide 29
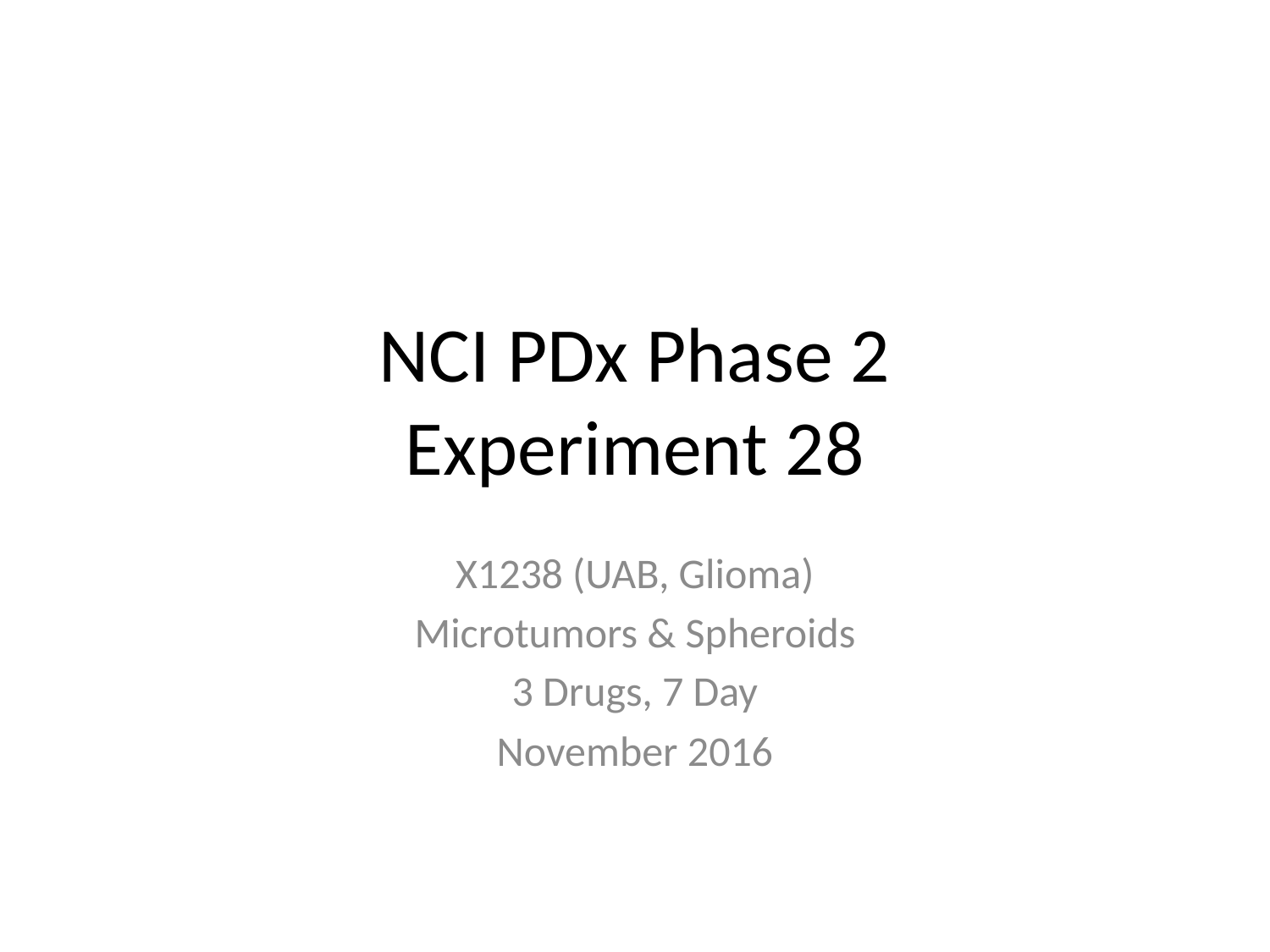

# NCI PDx Phase 2 Experiment 28
X1238 (UAB, Glioma)
Microtumors & Spheroids
3 Drugs, 7 Day
November 2016

## Slide 30
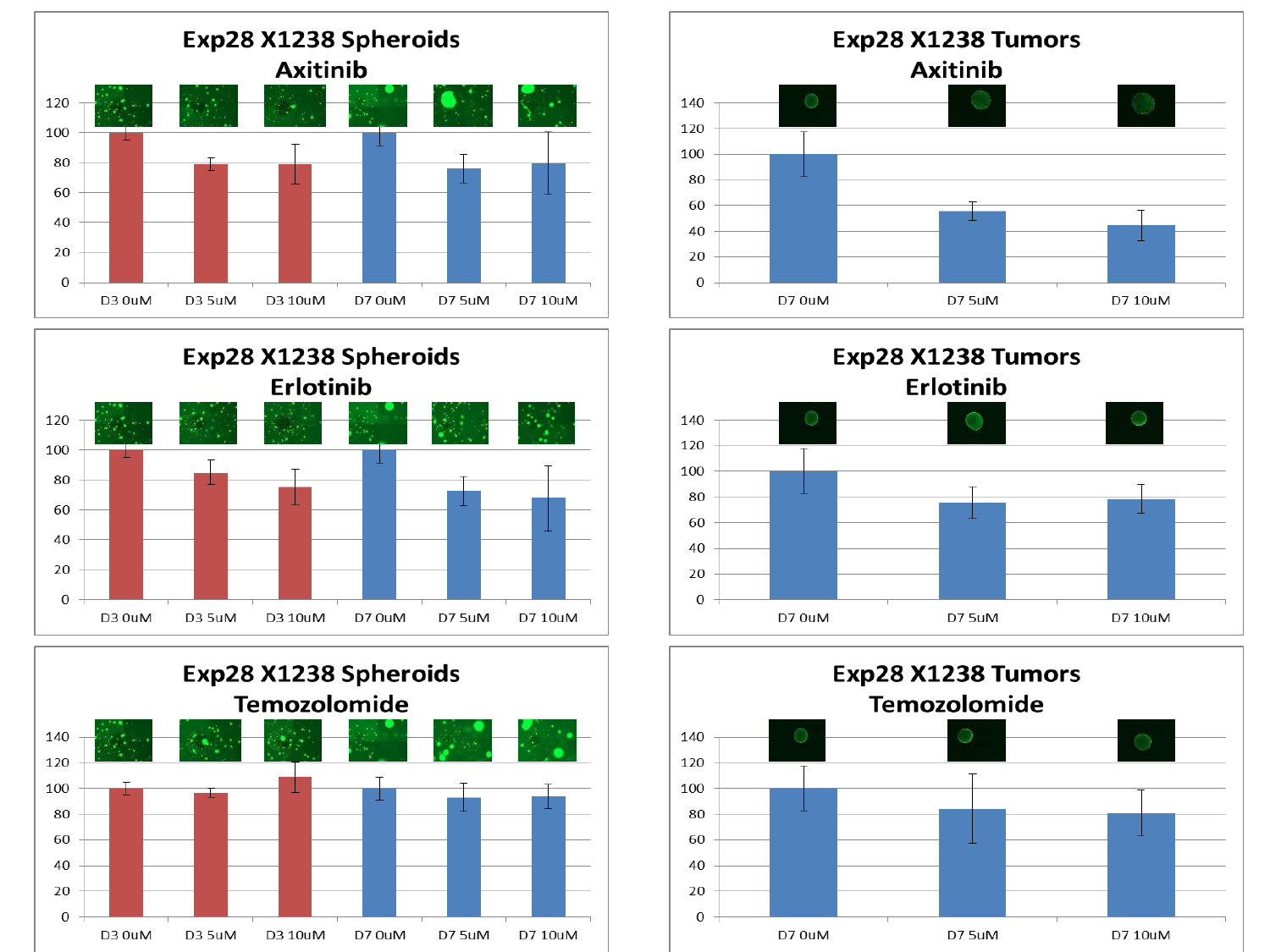

## Slide 31
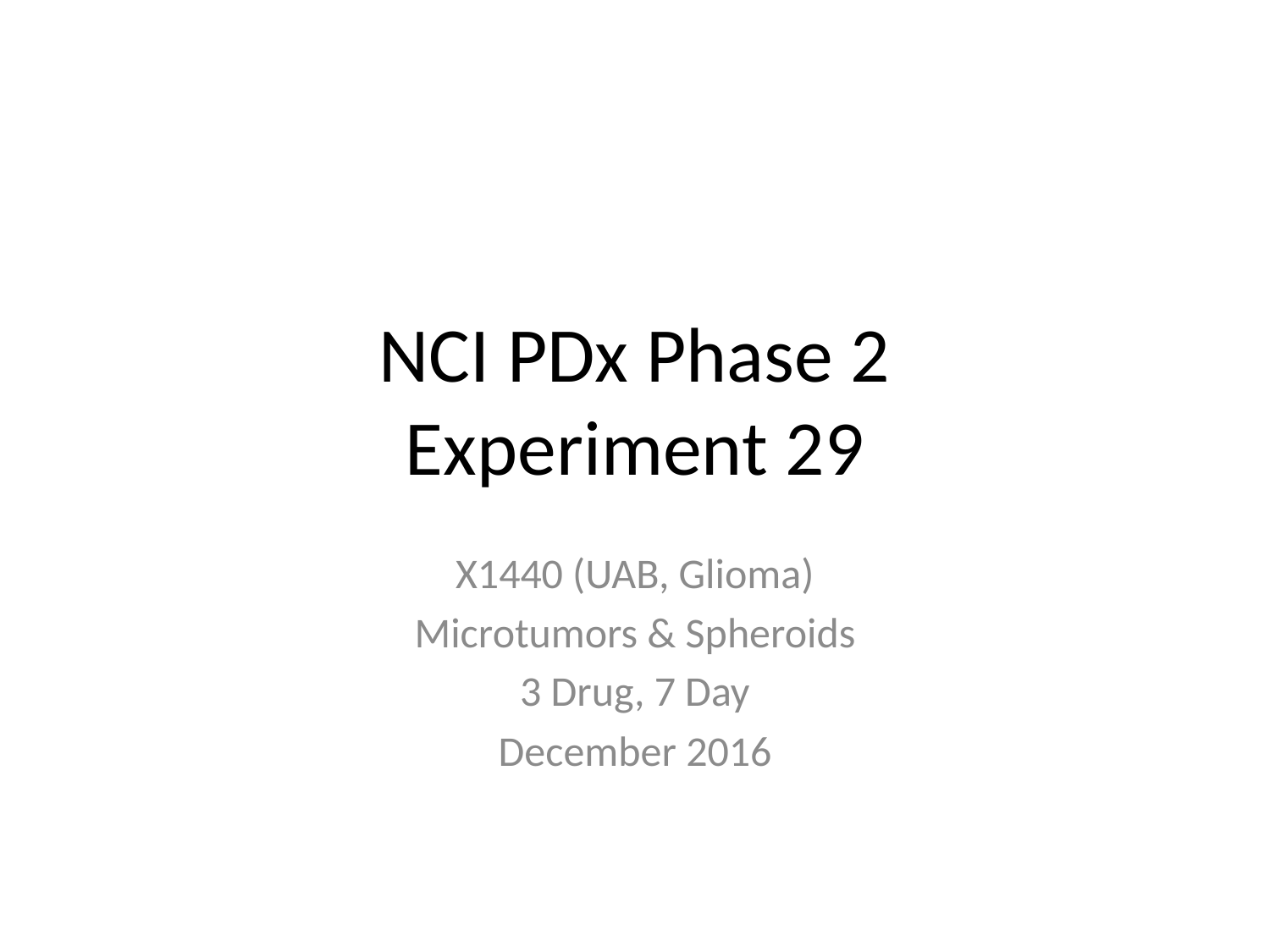

# NCI PDx Phase 2 Experiment 29
X1440 (UAB, Glioma)
Microtumors & Spheroids
3 Drug, 7 Day
December 2016

## Slide 32
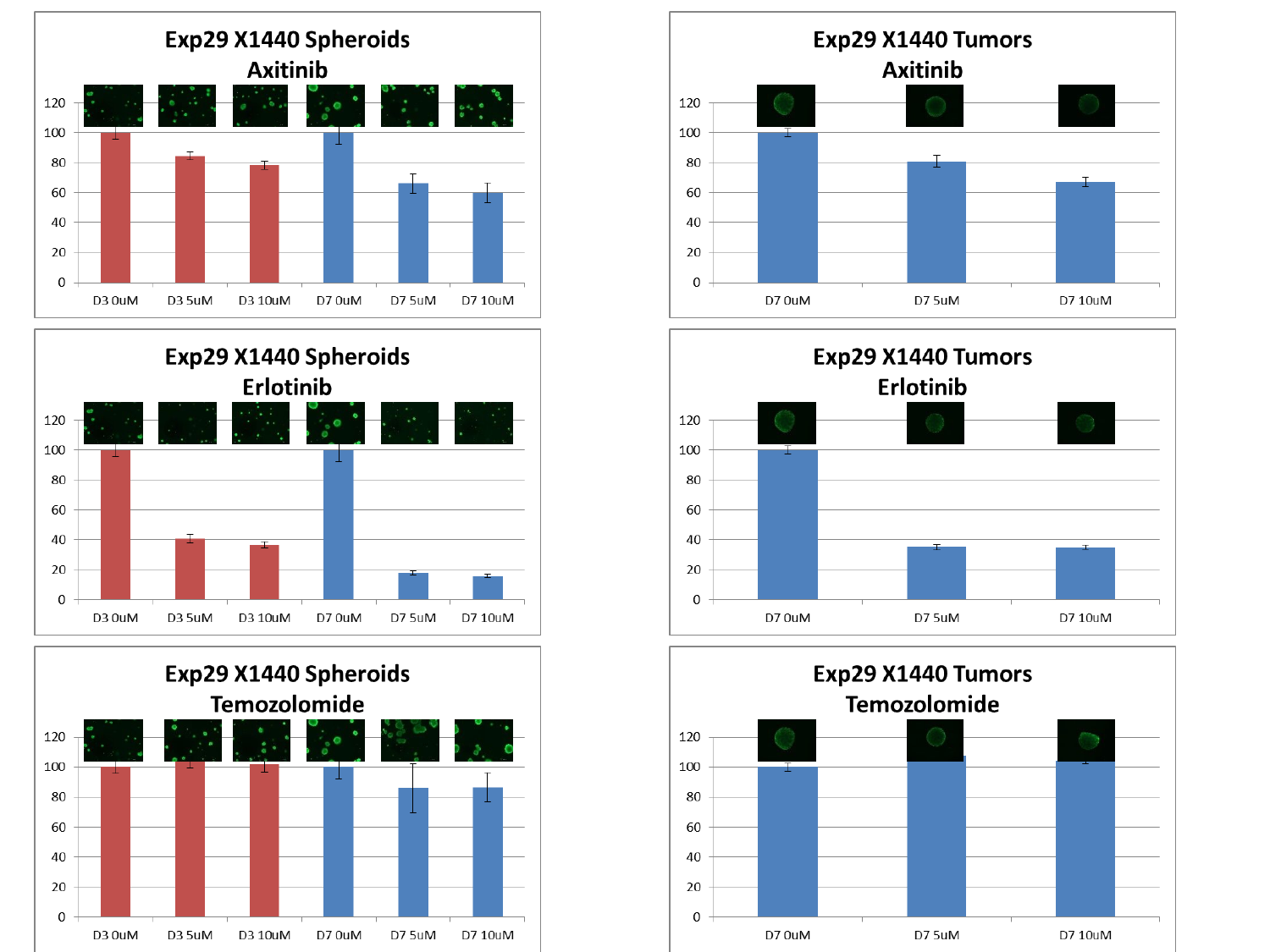

## Slide 33
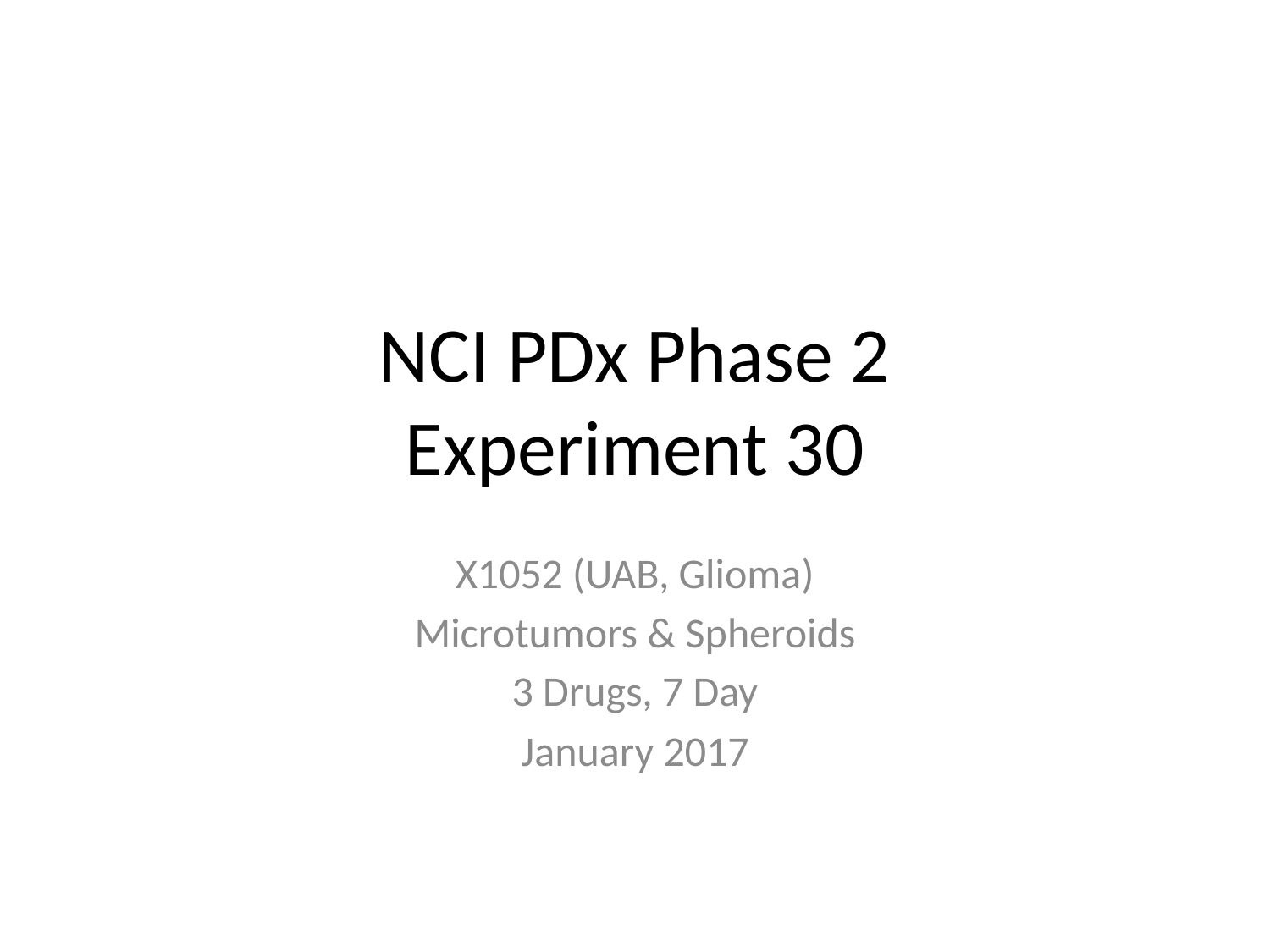

# NCI PDx Phase 2 Experiment 30
X1052 (UAB, Glioma)
Microtumors & Spheroids
3 Drugs, 7 Day
January 2017

## Slide 34
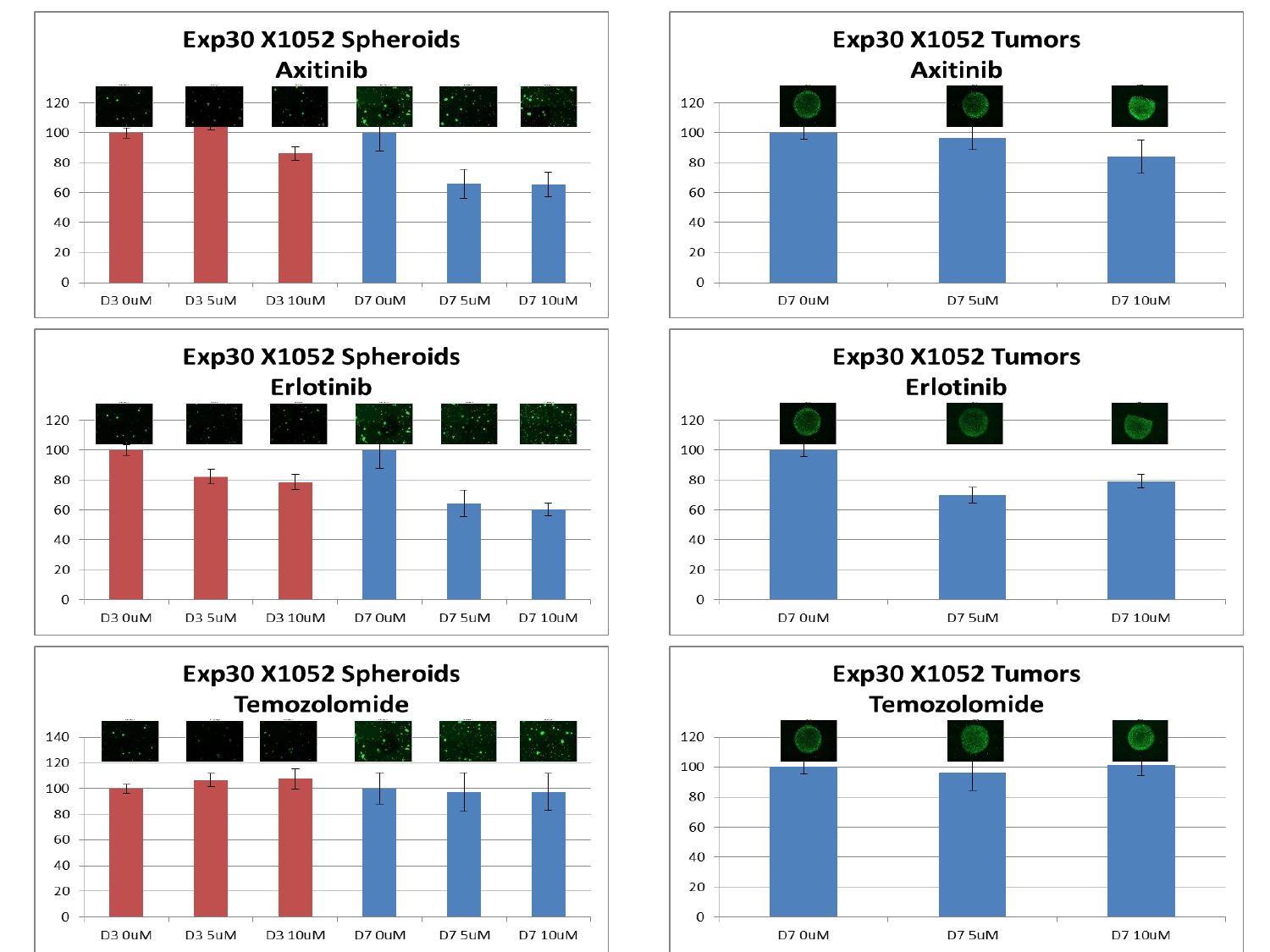

## Slide 35
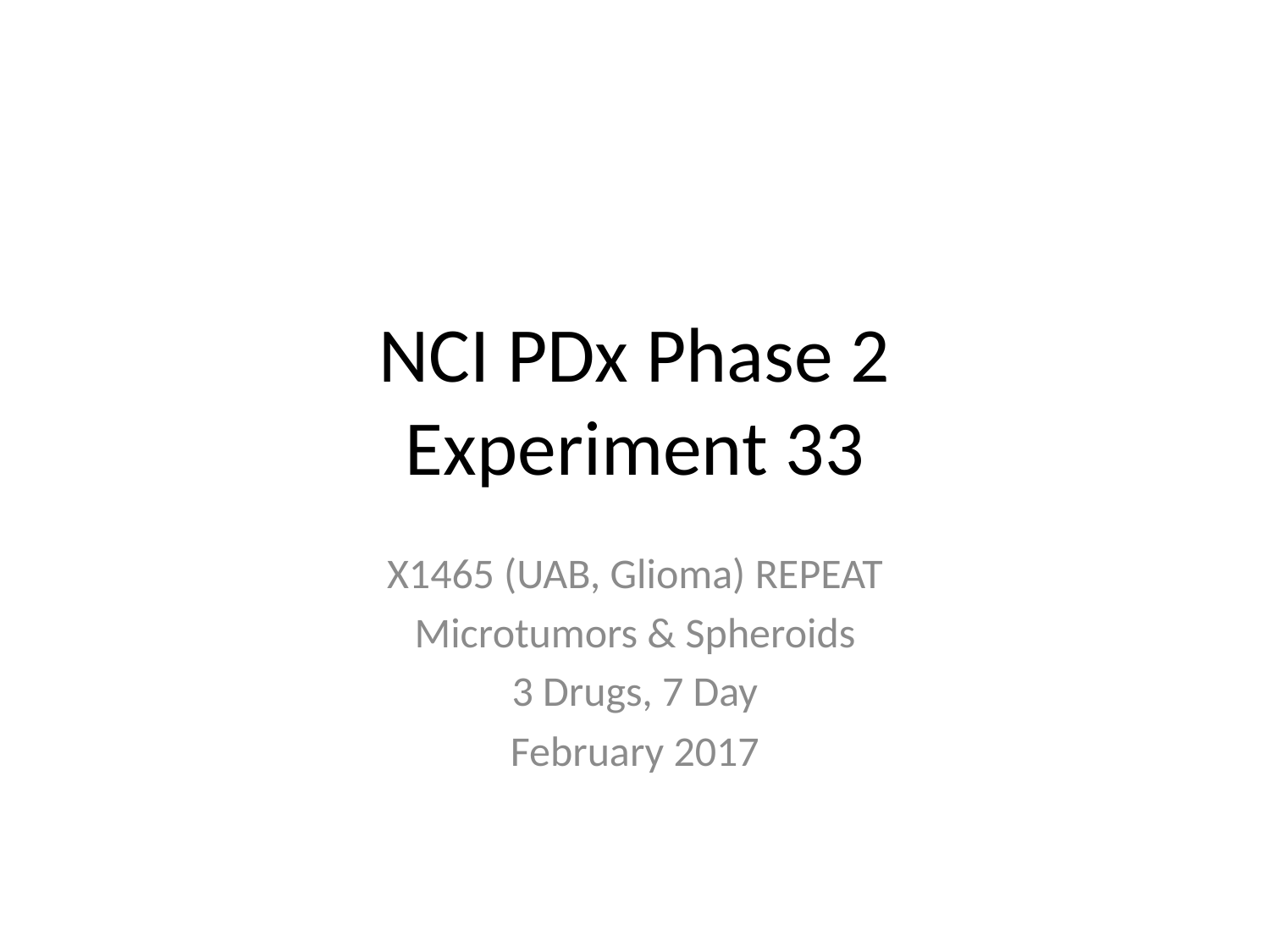

# NCI PDx Phase 2 Experiment 33
X1465 (UAB, Glioma) REPEAT
Microtumors & Spheroids
3 Drugs, 7 Day
February 2017

## Slide 36
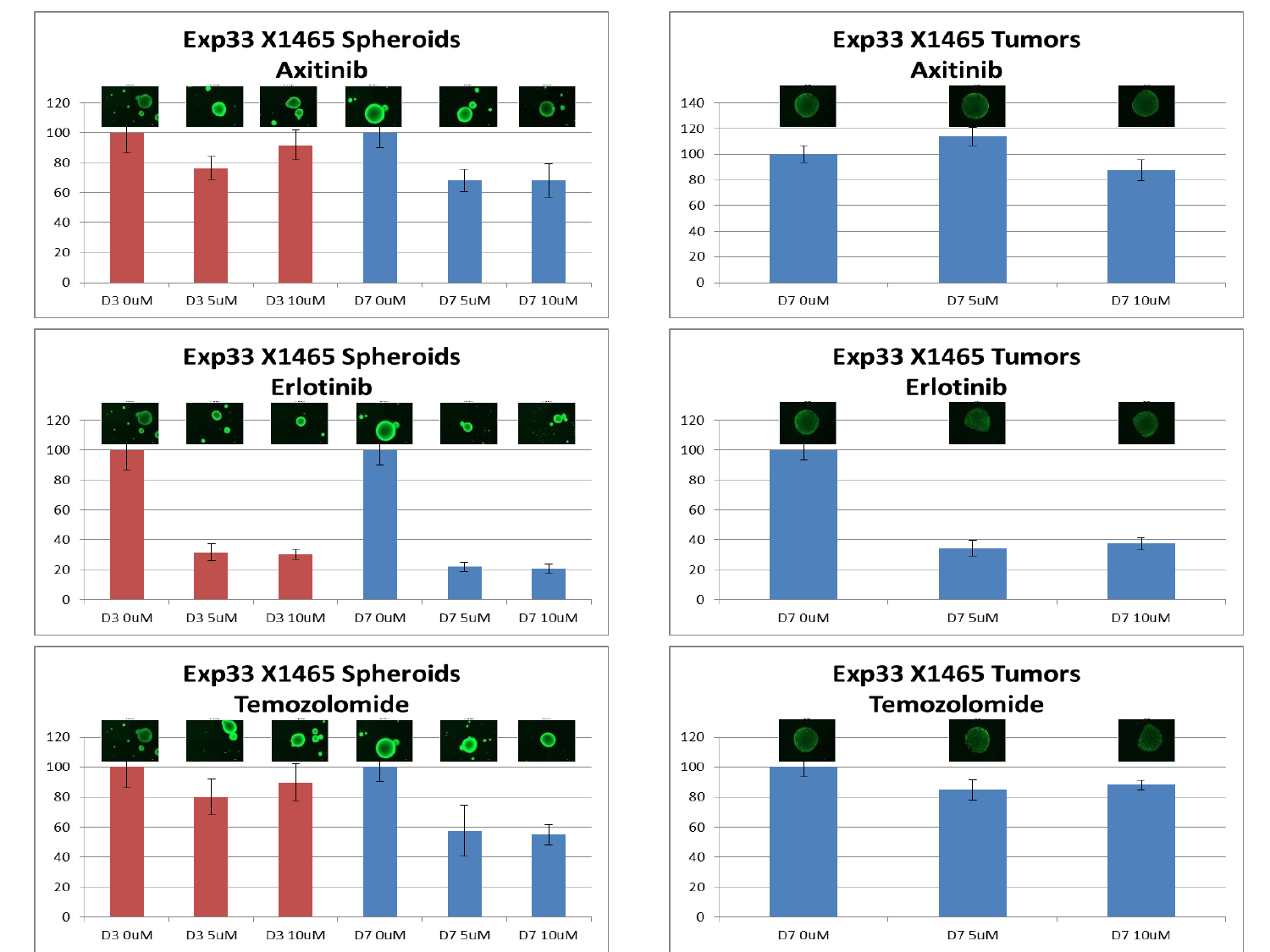

Supplement: Supplementary file 1 [file cells-08-00702-s001.zip › Supplementary File 1.pptx]
