# Supplementary figures and images for: A Novel Assay for Profiling GBM Cancer Model Heterogeneity and Drug Screening
Source: Cells. 2019 Jul 11;8(7):702. doi: 10.3390/cells8070702 (PMC6678976; doi:10.3390/cells8070702)

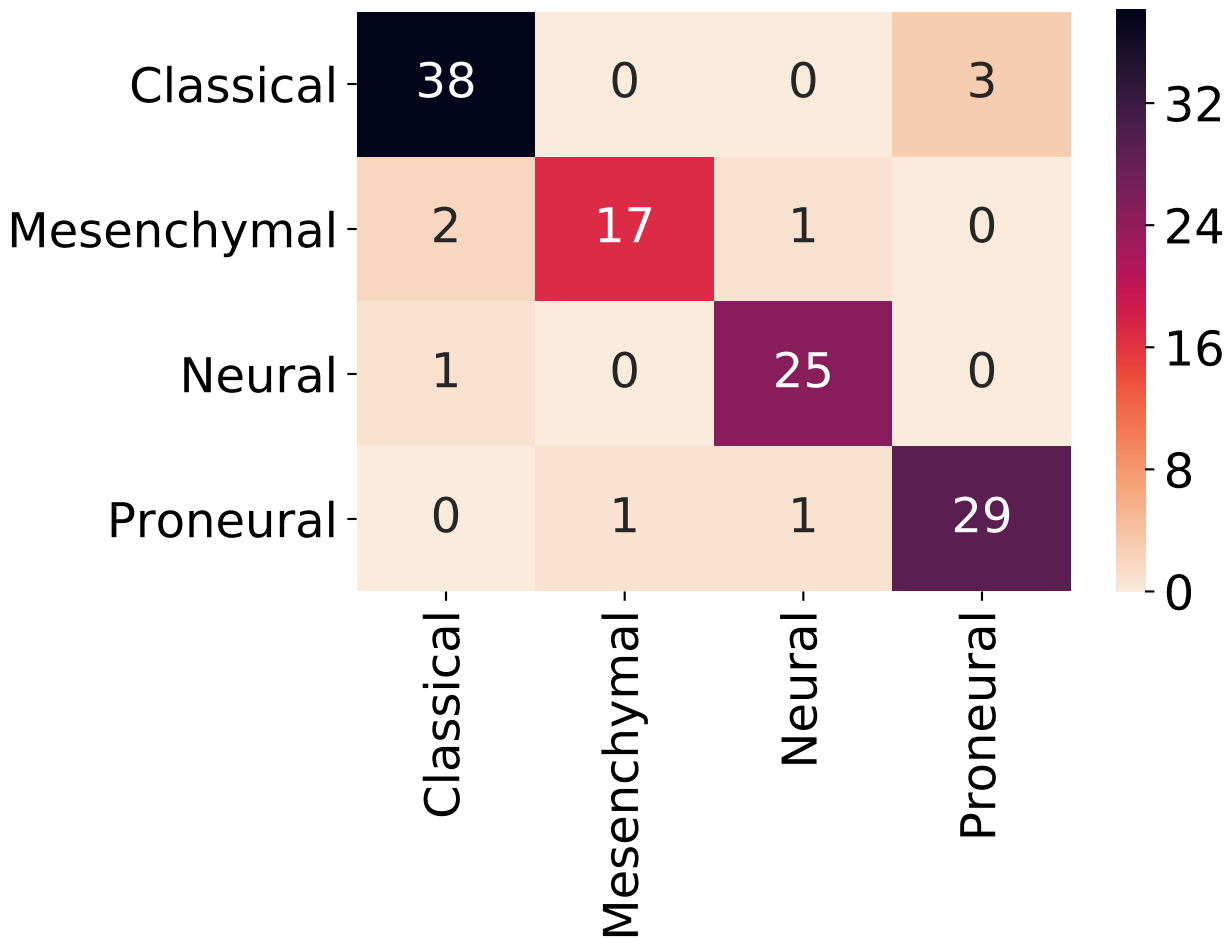

Supplement: Supplementary file 1 [file cells-08-00702-s001.zip › Figure S1.pdf]

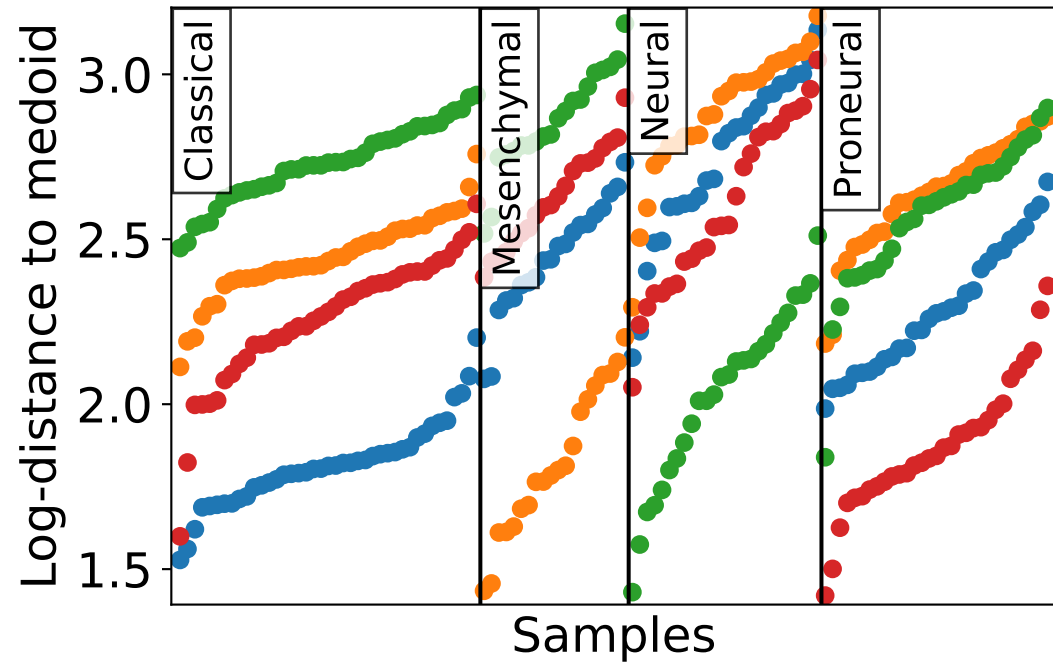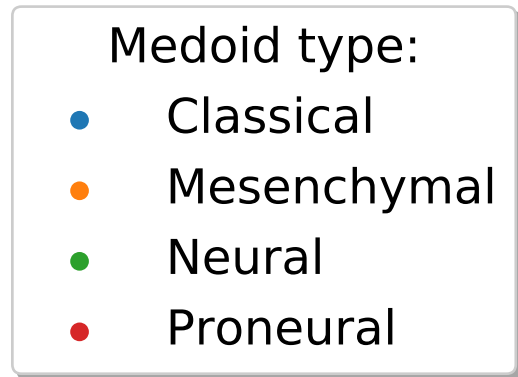

Supplement: Supplementary file 1 [file cells-08-00702-s001.zip › Figure S2.pdf]

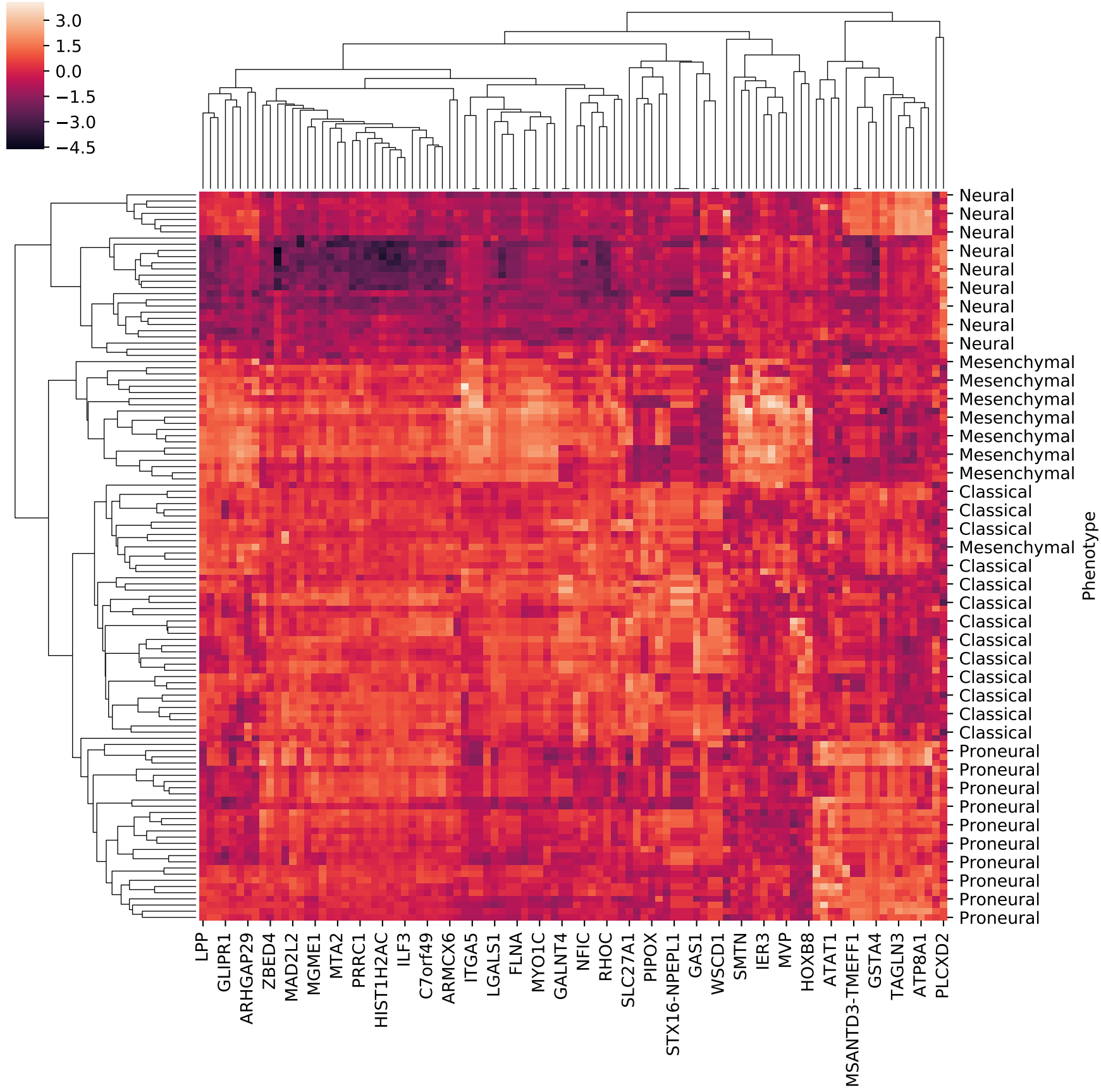

Supplement: Supplementary file 1 [file cells-08-00702-s001.zip › Figure S3.pdf]
